# Identification and Expression Analysis of Acid Phosphatase Gene (*PAP*) in *Brassica napus*: Effects of *cis*-Acting Elements on Two *BnaPAP10* Genes in Response to Phosphorus Stress

**DOI:** 10.3390/plants14030461

**Published:** 2025-02-05

**Authors:** Hongyuan Du, Ruiqian Zhang, Qingxue Zhang, Xun Shi, Jiaxue Wang, Qian Peng, Asfa Batool, Shisheng Li

**Affiliations:** 1 Hubei Key Laboratory of Economic Forest Germplasm Improvement and Resources Comprehensive Utilization, Huanggang 438000, China; pengqian114@mails.ucas.ac.cn (Q.P.); batool@lzu.edu.cn (A.B.); shishengli@hgnu.edu.cn (S.L.); 2 College of Biology and Agriculture Resources, Huanggang Normal University, Huanggang 438000, China; zuaaa1944984401@163.com (R.Z.); zhangqingxue031101@163.com (Q.Z.); sx13581284680@163.com (X.S.); 15034270048@163.com (J.W.)

**Keywords:** *Brassica napus*, purple acid phosphatases (PAP), expression analysis, phosphate stress, salt stress, *cis*-element

## Abstract

Purple acid phosphatases (PAPs) play a key role in phosphorus (P) assimilation and redistribution in plants, catalyzing the hydrolysis of phosphate esters to produce inorganic phosphate (Pi). In this study, a total of 77 *PAP* genes were identified in *B. napus*. The candidate genes were divided into three groups and ten subgroups based on the phylogenetic analyses and exon-intron organization. Among these 77 BnaPAP proteins, 35 exhibit typical metal-ligating residues characteristic of known PAPs, whereas certain unaltered amino acid residues were absent or displaced in other BnaPAPs. A computational prediction was conducted, revealing that the majority of PAPs contain signal peptide motifs and display a range of N-glycosylation levels, as well as transmembrane helix motifs. An analysis of previously obtained RNA-seq data revealed that 55.84% (43 of 77) of the *BnaPAPs* responded to Pi deficiency. Moreover, we conducted a preliminary examination of the expression profiles of *BnaPAP* genes in response to salt stress, and discovered that 42.86% (33 of 77) of these genes were induced under salt stress, either in the shoots or in the roots. Further qRT-PCR and GUS analyses revealed that *BnaC9.PAP10* and *BnaA7.PAP10*, two paralogs of *BnaPAP10s*, were induced by Pi deficiency. Notably, *BnaC9.PAP10* exhibits robust induction, compared to the relatively mild induction observed in *BnaA7.PAP10*. Our research shows that *BnaA7.PAP10* uniquely responds to Pi stress via the W-box, while *BnaA7.PAP10* predominantly responds via the P1BS element, and the differences in *cis*-regulatory elements (CREs) within their promoter regions specifically contribute to their distinct expression levels under Pi stress. Our findings provide valuable insights and establish a foundation for future functional studies of *BnaPAPs*.

## 1. Introduction

Plants primarily obtain P, an essential, yet frequently limiting, macronutrient, via their roots by absorbing inorganic phosphate (Pi) from the soil [1,2]. In most cultivated soils, the concentration of inorganic P is far below the minimum level required for plant growth. Plants have evolved numerous morphological, physiological, molecular, and biochemical adaptations to endure low Pi stress [3,4,5,6,7,8]. One evolutionary mechanism is the synthesis and secretion of acid phosphatases (APases) [9,10]. As a vital hydrolase, APases have emerged as a significant indicator of plant adaptation to low Pi stress. Purple acid phosphatase (PAP), a specific category of acid phosphatase (APase) in plants, has been extensively studied for its crucial role in the plant’s adaptation to Pi stress [11,12].

The distinctive purple or pink hue of PAPs in solution, which gives them their colorful nomenclature, arises from a charge transfer transition occurring at approximately 560 nm, involving the metal-coordinating tyrosine transitioning to the metal ligand Fe(III) [13]. PAPs belong to the metallophosphoesterase family, which consists of binuclear metal-containing acid hydrolases. These enzymes are ubiquitous in animals, plants, bacteria, as well as fungi [14]. PAPs hydrolyze a wide range of phosphomonoester and amide substrates [15]. All members of the PAP family contain seven highly conserved amino acid residues that serve as metal ligands, located within five motifs at their carboxyl termini: (**D**XG/G**D**XX**Y**/G**N**H(D/E)/VXX**H**/G**H**X**H** (The conserved amino acid residues are emphasized with bold and underlined characters), which are required for the formation of the enzyme’s binuclear center [16]. Traditionally, plant PAPs have been categorized into two primary groups based on their molecular weight: LMW (low molecular weight) PAP (about 35 kD) and HMW (high molecular weight) PAP (about 55 kD). HMW PAPs typically exist as homodimers and share homology with enzymes from fungi and mycobacteria, whereas LMW PAPs primarily exist in monomeric form and are more closely related to their mammalian counterparts [11,12,16]. Plant PAPs have been categorized into three groups based on their predicted protein sequences: Group I and II encompass HMW oligomeric PAPs, while Group III consists of LMW monomeric PAPs [17]. The majority of PAPs are secretory proteins that exhibit diverse subcellular locations and possess the ability to hydrolyze phosphate esters, including ADP, ATP and glycolipids. Consequently, plant PAPs primarily function in the acquisition and utilization of inorganic Pi [12,18]. Additionally, they have been implicated in various other processes, including cell wall biosynthesis [19], salt stress responses, and carbon and nitrogen metabolism [20,21,22,23,24].

*PAP* genes have been identified in numerous plant species [16,25,26,27,28,29,30,31]. In *Arabidopsis*, there are 29 *PAP* members, and at least 11 of these *PAP* genes are upregulated in response to Pi starvation [16]. Among them, *AtPAP10*, *AtPAP12*, *AtPAP15*, *AtPAP17* and *AtPAP26* have been well-characterized [32]. AtPAP10 is an APase induced by Pi starvation, predominantly localized to the root surface, which exhibits phosphatase activity towards a range of substrates [33,34]. The deletion of AtPAP10 leads to a decrease in root-associated phosphatase activity by approximately 40%, whereas its overexpression enhances the plant’s adaptive capabilities under Pi stress conditions [33]. AtPAP12 and AtPAP26 are the two of the most important secreted APases, that enhance Pi acquisition by breaking down soil-bound organophosphates. Although *AtPAP26* has an important function in response to Pi stress, its expression is not induced by low Pi conditions [35]. *AtPAP17 (AtACP5),* an LMW PAP, was the first phosphate-starvation-induced (PSI) PAP characterized in *Arabidopsis* under Pi deficiency [36]. Recent research has also indicated that AtPAP17 and AtPAP26, two novel purple acid phosphatases, are associated with a high salt tolerance under NaCl stress conditions [22]. The overexpression of *AtPAP15*, fused with a carrot extracellular, targeting peptide in soybean plants, has been shown to enhance phytase activity, leading to significant improvements in growth and P-utilization efficiency of the transgenic plants grown in sand with phytate as the sole P source [37]. In rice, overexpressing *OsPAP10a*, *OsPAP10c*, and *OsPAP21b* boosts APase activity and organic phosphate degradation [38,39,40].

The allotetraploid *Brassica napus* (2n = 4x = 38, AnAnCnCn) emerged ~7500 years ago through the natural crossing of *Brassica rapa* (2n = 20, ArAr) and *Brassica oleracea* (2n = 18, CoCo) [41]. Rapeseed as a crop requires substantial amounts of Pi fertilizer and is particularly susceptible to the effects of P deprivation [42]. In addition, as the problem of soil salinization intensifies globally, soil salinity constitutes a pivotal environmental constraint on plant growth and development, and notably diminishes rapeseed yield under salt stress [43]. Despite extensive research on *PAPs* and their roles in plant adaptation to Pi stress [16,25,32,33,34,35,36,37,38,39,40], there have been few comprehensive functional analyses of *PAPs* in *Brassica napus* [44,45,46], especially when it comes to studying the *BnaPAPs* genes under salt stress conditions. In this research, we conducted a comprehensive examination of the *PAP* gene family in *B. napus* on a global scale, accompanied by a series of bioinformatic assessments of the candidates, including gene number, phylogenetic relationship, motifs, collinearity relationship, and expression profiling of *BnaPAP* genes in response to Pi deficiency and salt stress. Ultimately, we examined the CREs in *BnaC9.PAP10* and *BnaA7.PAP10*, which exhibit notable variations in induction folds when subjected to Pi stress. Our findings confirm that the distinct expression patterns of these two genes under Pi stress are attributed to differences in the CREs located within their promoter. Our work laid the basis for further studies of the biological function of *PAP* genes and the genetic improvement in *B. napus*.

## 2. Results

### 2.1. Identification and Phylogenetic Analysis of PAP Family Genes in B. napus

**Figure 1:**
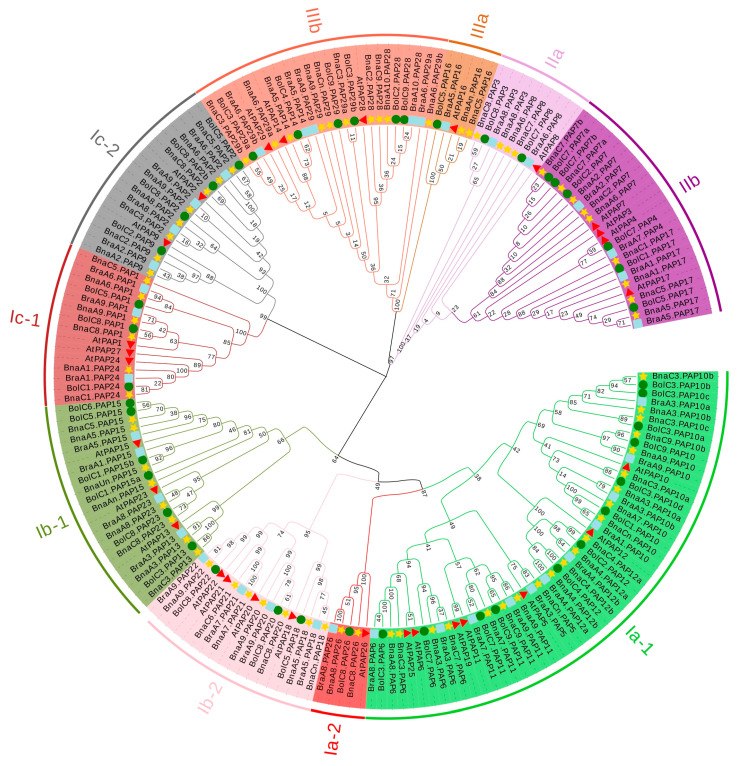
Phylogenetic analysis of *PAP* members in *B. napus, B. rapa, B. oleracea* and *A. thaliana*. The ten subgroups were distinguished by different colors. The NJ tree was generated using ClustalW in MEGA7 using the full-length amino acid sequences of the *B. napus* PAP proteins (yellow star), *B. rapa* PAP proteins (blue square), *B. oleracea* PAP proteins (green circle) and AtPAPs (red triangle).

Based on the homology of the 29 *PAPs* in *Arabidopsis*, a total of 77 *PAP* genes were identified in the whole genome of *B. napus*. Among these, 35 BnaPAP proteins exhibit the presence of five conserved motif blocks (G**D**XG/G**D**XX**Y**/G**N**H(D/E)/VXX**H**/G**H**X**H**), as well as seven conserved residues that are crucial for the ligation of the dimetal nuclear center, a feature that is a characteristic of the known PAPs (Table S1). In the case of BnaPAP1s, BnaPAP2s, BnaPAP9s, and BnaPAP24s, the “Y” residue within the second conserved motif block (G**D**XX**Y**) is substituted by “K”, “N”, or “S.” Conversely, the rest of the BnaPAPs are deficient in one or more of these conserved blocks. These genes are also classified as *PAP* genes due to the significant homology in their amino acid sequences with those of known *PAPs*. A neighbor-joining (NJ) phylogenetic tree was constructed based on the multiple alignments of the amino acid sequences of BnaPAPs, BraPAPs (39), BolPAPs (44), and AtPAPs (29) (Figure 1). The results showed that all PAP proteins were classified into three major groups (I to III), and a further classification of these three major groups produced ten subgroups (Ia-1, Ia-2, Ib-1, Ib-2, Ic-1, Ic-2, IIa, IIb, IIIa and IIIb). Among the three groups, Group I harbored the highest number of PAPs, comprising 128 members (specifically, 20 AtPAPs, 52 BnaPAPs, 29 BolPAPs, and 27 BraPAPs). Notably, the majority of the identified Pi-associated PAPs are localized within this group. Group II and III contained 32 (4 AtPAPs, 13 BnaPAPs, 9 BolPAPs and 6 BraPAPs) and 29 members (4 AtPAPs, 12 BnaPAPs, 7 BolPAPs and 6 BraPAPs), respectively. The *BnaPAPs* were closely clustered with their corresponding homologs in *A. thaliana,* and each *AtPAP* was related to zero to nine *BnaPAP* homologs. The number of *PAP* homologs in *B. napus* was comparable to the combined total of *PAPs* in the genomes of *B. rapa* and *B. oleracea*, suggesting that the majority of *PAPs* were retained during the hybridization event. We also examined the count of *PAP* genes across 17 whole-genome sequenced plant species, and found that the copy number of *PAPs* in *B. napus* was the largest among the 17 species; furthermore, there seems to be no strong correlation between the number of *PAPs* and the size of the plant genome (Figure S1).

### 2.2. Molecular Characterization Analysis of BnaPAP Proteins

**Figure 2:**
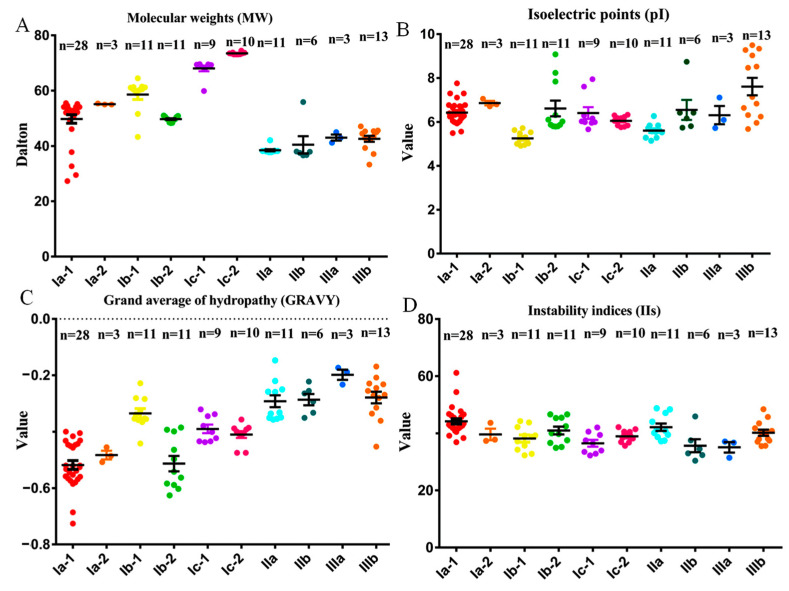
Molecular characterization of BnaPAP proteins. (**A**–**D**), Molecular weights (MWs, (**A**)), theoretical isoelectric points (pIs, (**B**)), grand average of hydropathy (GRAVY, (**C**)) values and instability indices (IIs, (**D**)) of the BnaPAP proteins. The GRAVY value is defined as the sum of hydropathy values of the amino acids divided by the protein length. An II value < 40.0 indicates the stability of protein.

The deduced BnaPAP proteins were analyzed for their general information, including the predicted size, presence of signal peptides, subcellular location, and potential N-linked glycosylation sites (Figure 2, Table S2). The molecular weights (MWs) of the identified PAPs ranged from 29.5 kDa (BnaCn.PAP5) to 74.5 kDa (BnaC3.PAP2), which corresponded to a variation in the number of deduced amino acids (AAs) from 265 to 663. EXPASY analysis revealed that most PAP proteins in the same subgroup had similar parameters (Figure 2). Group I is almost entirely composed of HMW PAPs (about 55 KD), and group II and III are composed of LMW mammalian-like PAPs (about 40 KD) (Figure 2A). The theoretical isoelectric points (pIs) of the BnaPAP proteins ranged from 4.91 (BnaA3.PAP13) to 9.34 (BnaCn.PAP29), with a notable exception of six BnaPAPs, and nearly all of the remaining ten subgroups exhibited pIs below 7.0 (Figure 2B). The GRAVY values among the BnaPAP members varied, with the lowest being −0.726 for BnaA7.PAP10, and the highest being −0.188 for BnaAn.PAP16 (Figure 2C). Therefore, all BnaPAPs in the ten subgroups were presumed to be hydrophilic. The majority of BnaPAPs had instability index (II) values of less than 40.0, as illustrated in Figure 2D, suggesting a robust stability profile for these proteins. Using SignalP 3.0, it was predicted that 63 BnaPAP proteins possessed a signal peptide, while TargetP 1.1 predicted that 69 of them were secretory proteins. An analysis of the N-glycosylation site prediction revealed that BnaPAPs have varying numbers of glycosylation sites, ranging from 0 to 8. When the transmembrane structures of BnaPAPs were characterized using the TMHMM tool, it was found that BnaPAPs contained 0 to 2 membrane-spanning regions. Specifically, 40 BnaPAPs had one transmembrane region, one BnaPAP (BnaA2.PAP9) had two membrane-spanning regions, and the remaining 36 BnaPAPs lacked any transmembrane region (Table S2).

### 2.3. Chromosomal Localization and Gene Structure of the Identified BnaPAPs

**Figure 3:**
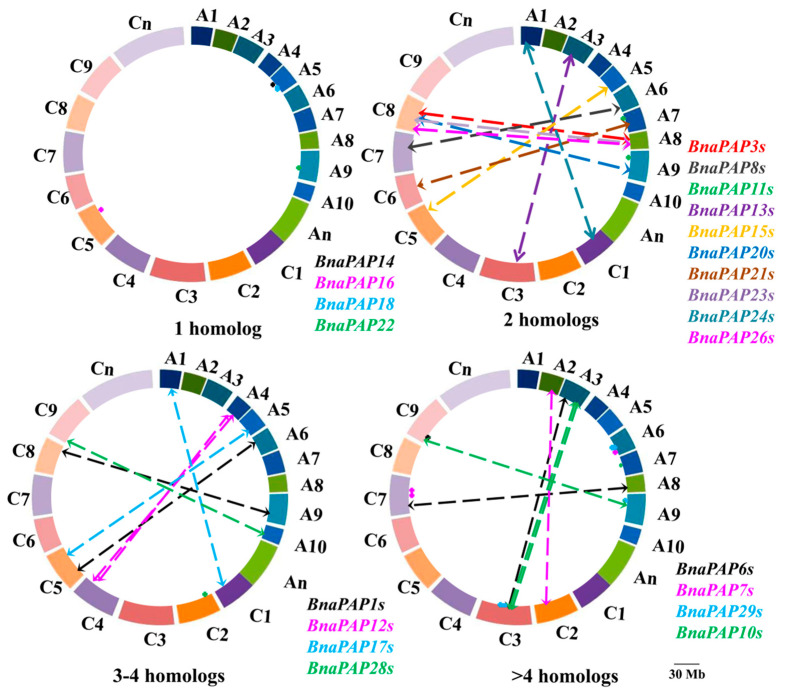
Physical mapping and syntenic analysis of *PAP* family genes in *B. napus*. The *BnaPAP* homologs between the An and Cn subgenomes of rapeseed are connected by crashed lines. The length scale of *B. napus* chromosomes (An subgenome: A1–A10; Cn subgenome: C1–C9) is 30.0 Mb.

**Figure 4:**
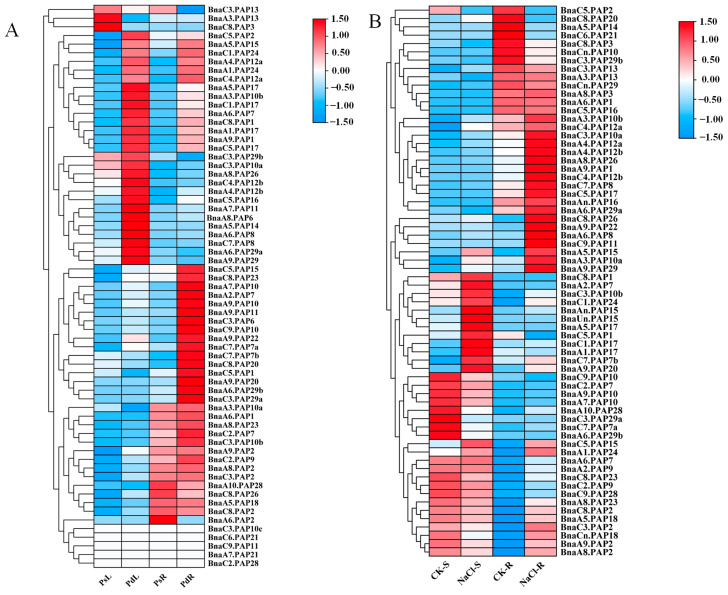
The expression patterns of the *PAP* gene family in *B. napus* under Pi stress and NaCl stress conditions were analyzed by RNA-seq. The color scale is indicated on the right. The fragments per kilobase of transcript per million mapped reads (FPKM) data (**A**) and the transcripts per million (TPM) data (**B**) were standard transformed into their log2-scaled expression values, which were then utilized to generate the heatmap. PsL: P-sufficient leaves; PdL: Pi-deficient leaves; PsR: Pi-sufficient roots; PdR: Pi-deficient roots. CK-S: Control Shoots; NaCl-S: NaCl-stress Shoots; CK-R: Control Roots; NaCl-R: NaCl-stress Roots.

Chromosomal location analysis indicated that 76 *BnaPAP* genes (excluding *BnaUn.PAP15*) were unevenly scattered across the 19 chromosomes of *B. napus*, with an equal distribution of 38 genes in both the A and C subgenomes (Figure 3). Six genes were positioned within the A and C subgenomes, but their exact chromosomal locations are still unknown. The number of *PAP* genes on a single *B. napus* chromosome range from 1 (A10, C06) to 8 (C03). In our study, we discovered that 15 *BnaPAP* genes, accounting for 19.48% of the total number, were grouped into six regions of tandem duplication on chromosomes A03, A04, A06, C03, C04, and C07 (Table S3). This finding indicates that a limited number of tandem duplication events have contributed to the expansion of the *PAP* gene family in *B. napus*. Furthermore, in addition to these tandem duplications, our analysis of the chromosomal gene location and homology synteny revealed that 57.14% (44 out of 77) of the *PAP* genes were the result of segmental duplication (Figure 4). These findings suggest that segmental duplication events were a significant factor in the expansion of the *BnaPAP* family. During the evolution of a multigene family, gene structure usually diversifies. With the exception of *BnaA2.PAP9* and *BnaC2.PAP9*, the majority of *BnaPAPs* possessed more than one intron, hinting at the potential occurrence of alternative splicing during gene transcription. The most prevalent organizational pattern involved seven exons interspersed with six introns, which was observed in 20 *BnaPAPs* (Figure S2).

### 2.4. Expression Patterns of BnaPAPs Under Pi Deficiency and Salt Stress

Previous studies have shown that some *PAP* genes are involved in salt stress tolerance [22]. To thoroughly examine the potential functions of *BnaPAP* genes, an RNA-seq assay was conducted on *Brassica napus* seedlings to investigate the expression profiles of the *BnaPAPs* in response to Pi deficiency and NaCl stress. The FPKM (Fragments Per Kilobase Million) values for 65 *BnaPAP* genes, obtained from the experimental data under Pi stress, were extracted and subsequently transformed into logarithmic scale representations, which were then displayed in a heatmap format (Figure 4A). Among them, 66.15% (43/65) were found to be significantly induced by Pi starvation, specifically, 16 *BnaPAP* genes (*BnaC5.PAP1, BnaC3.PAP6, BnaA2.PAP7*, *BnaC7.PAP7a-b, BnaA7.PAP10, BnaA9.PAP10, BnaC9.PAP10, BnaA9.PAP11, BnaC5.PAP15, BnaA9.PAP20, BnaC8.PAP20, BnaA9.PAP22, BnaC8.PAP23, BnaA6.PAP29b,* and *BnaC3.PAP29a*) exhibited strong induction specifically in the roots, with moderate or no significant changes in their expression levels in the shoots. Conversely, 13 *BnaPAP* genes, including members of the *BnaPAP10s*, *BnaPAP12s*, and *BnaPAP29s* subfamilies, were induced by Pi stress exclusively in the shoots, with minimal response in the roots. Additionally, 14 *BnaPAP* genes (*BnaA9.PAP1, BnaC8.PAP1, BnaC5.PAP2, BnaA6.PAP7, BnaA3.PAP10b, BnaA4.PAP12a, BnaC4.PAP12a, BnaA5.PAP15,* 4 *BnaPAP17s* and 2 *BnaPAP24s*) showed robust upregulation in both roots and shoots in response to Pi deprivation. (Figure 4A, Table S4). Three *BnaPAP* genes (*BnaA3.PAP13, BnaC3.PAP13,* and *BnaC8.PAP3*) exhibited decreased expression levels in response to Pi stress. Until now, there is limited information available regarding the expression patterns of the *BnaPAP* genes under salt stress. In this research, 66 *BnaPAPs* were detected to perform different expression levels under control (−NaCl) and salt (+NaCl, 200 mM NaCl) treatments (Figure 4B, Table S5). These *BnaPAPs* were clustered and divided into six groups. It is noteworthy that within these groups, 17 *BnaPAPs* were exclusively induced in the roots, whereas 12 were exclusively induced in the shoots by NaCl stress. *BnaA1.PAP24* was slightly induced both in the shoots and roots. Alternatively, the expression of eight genes—comprising 3 *BnaPAP10s*, 3 *BnaPAP7s*, 2 *BnaPAP29s*, and *BnaA10.PAP28*—was downregulated in the shoots. Additionally, six *BnaPAP* genes showed suppressed expression in the roots. Notably, *BnaPAP2* experienced downregulation in both the roots and shoots.

### 2.5. Differential Transcriptional Responses of BnaPAPs to Pi Deprivation by qRT-PCR

**Figure 5:**
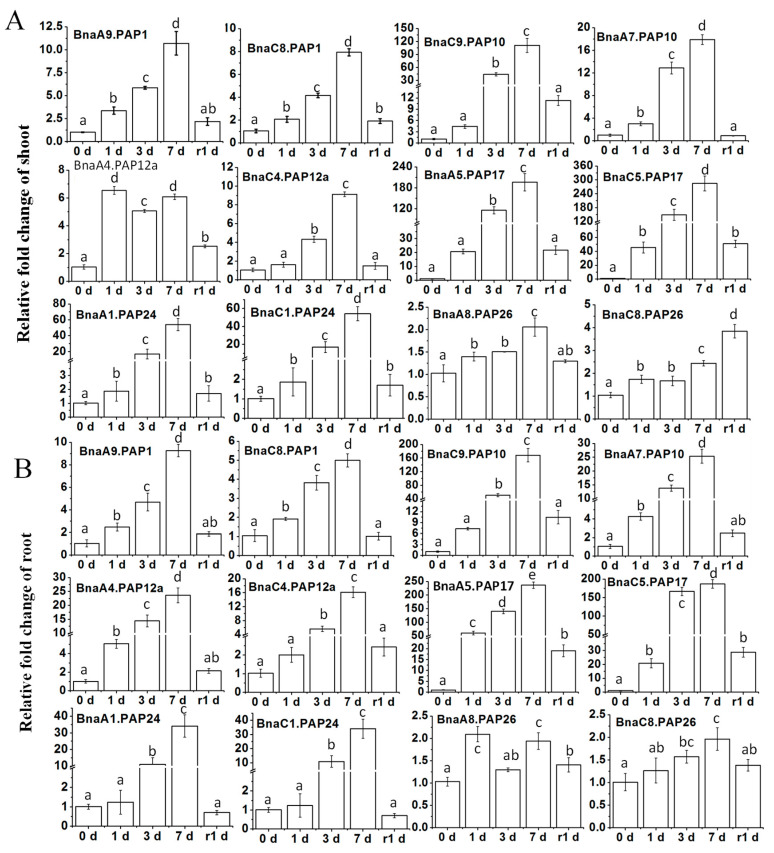
The expression changes of 12 *BnaPAP* genes under Pi deficiency and recovery in *B. napus*. Fifteen-day-old plants were subjected to Pi starvation (0 μM Pi) for seven days, followed by Pi replenishment (RP). Samples were collected from shoots and roots at five different time points: initially (0 d), and then at 1 d, 3 d, and 7 d after Pi starvation, as well as 1 d after RP. qRT-PCR was utilized to quantify transcript levels of *BnaPAPs* in shoots (**A**) and roots (**B**). Data presented are ratios of signal intensity at each time point to signal intensity at the baseline (0 d). Values represent mean ± standard deviation (SD) of three biological replicates with three plants each. Different lowercase letters on the bars indicate significant differences (*p* < 0.05; Tukey’s test; n = 3).

To gain a comprehensive understanding of the expression patterns of *BnaPAPs* during the Pi starvation and the subsequent recovery, qRT-PCR was conducted using specific primers targeting 12 selected *BnaPAP* genes. The expression patterns of all selected genes at the 7 day mark closely mirrored the initial RNA-Seq findings, despite minor discrepancies in the fold-change of expression (Figure 4A and Figure 5). Further analysis of the temporal expression profiles of these particular *BnaPAPs* in both shoots (Figure 5A) and roots (Figure 5B) revealed that, apart from *BnaPAP26s*, most genes were upregulated within the first day following the Pi stress (one DPP) and sustained high expression levels until three DPP, peaking at seven DPP. Notably, the increased *BnaPAP* transcripts were promptly downregulated after one day of Pi replenishment. Conversely, the transcript abundance of *BnaPAP26s* remained largely unaffected by low Pi conditions, despite being expressed at a relatively high level (Figure 5). Furthermore, the results indicated that certain paralogous genes exhibited comparable expression patterns and abundances, such as *BnaA9.PAP1* and *BnaC8.PAP1*, *BnaA5.PAP17* and *BnaC5.PAP17*, as well as *BnaA1.PAP24* and *BnaC1.PAP24*. However, some paralogous genes, such as *BnaA7.PAP10* and *BnaC9.PAP10*, exhibit similar stress response patterns but differ significantly in their fold-change induction levels. In both roots and shoots, *BnaC9.PAP10* demonstrates a higher fold-change induction compared to *BnaA7.PAP10*, with a nearly 10-fold difference. In aggregate, these findings indicate that the *BnaPAP* genes exhibit diverse transcriptional profiles in response to the Pi deficiency. qRT-PCR was also employed to investigate the expression patterns of these 12 *BnaPAPs* across various tissues—specifically, old leaves (OL), young leaves (YL), flowers, pods, and peduncles—in response to Pi deficiency (Figure S3). *BnaPAPs* showed elevated expression during Pi deficiency, with distinct patterns among individual genes. Specifically, *BnaA9.PAP1* was abundant in pods and peduncles, and *BnaC8.PAP1* in flowers, while *BnaA4.PAP12a* was abundant in reproductive organs, and *BnaC4.PAP12a* was abundant in young leaves. Of note, *BnaPAP26s* were unresponsive at the seedling stage but expressed higher in pods (Figure S3).

### 2.6. Characterization of Putative CREs in the Promoter Regions of BnaPAPs

**Figure 6:**
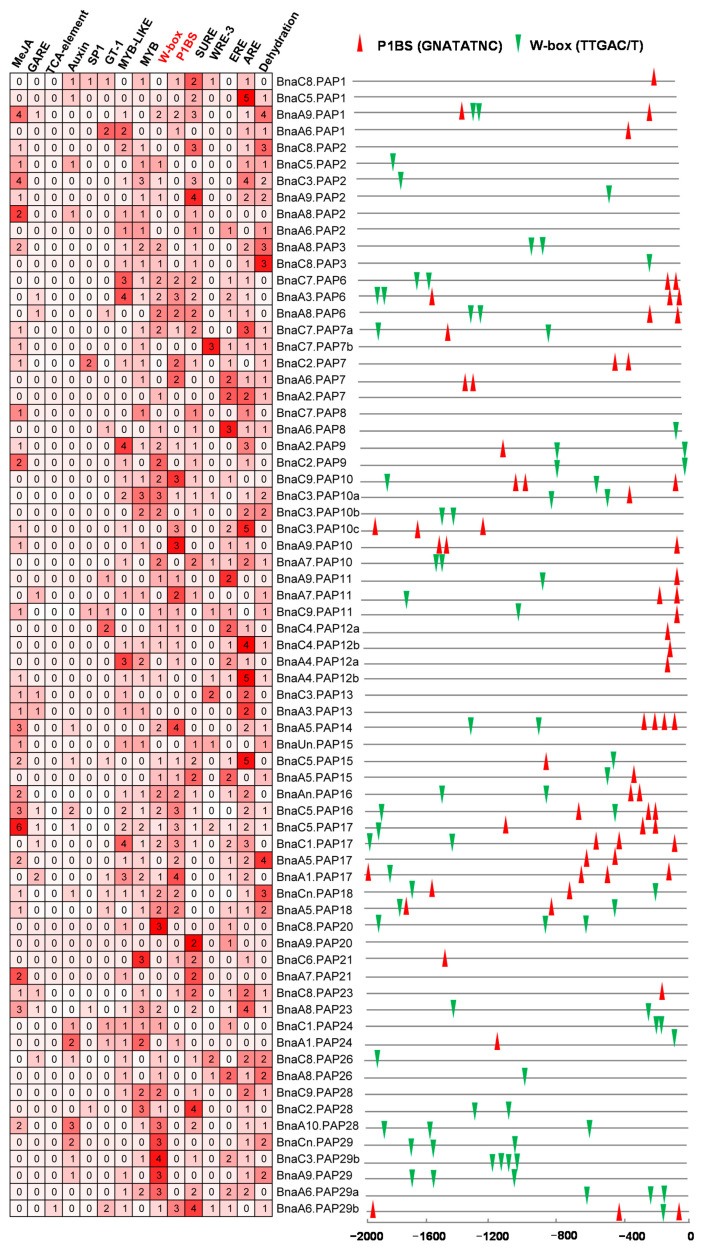
The distribution of CREs in promoters of *BnaPAP* family members. Different colors and numbers indicate numbers of different CREs in these *BnaPAP* genes, larger numbers correspond to darker red squares, indicating a higher count of the *cis*-element within gene promoters (**left**). P1BS (red triangle) and W-box (green triangle) are represented. The two-colored triangle represented P1BS and W-box, respectively, and their locations in each *BnaPAP* gene (**right**).

The *cis*-regulatory element (CRE) exerts a crucial influence on the control of gene expression. The plants’ responses to Pi deficiency involve intricate transcriptional regulation, which encompass multiple interconnected molecular signaling pathways. At the core of Pi signaling is PHR1, a pivotal regulator that controls the expression of downstream genes via the P1BS element (GNATATNC) [47,48]. Furthermore, emerging evidence underscores the significance of WRKY proteins in the Pi stress response, as they specifically interact with the W-box (TTGACC/T) motifs found in the promoters of target genes [49]. The GT-1 *cis*-element (GAAAAA) has been identified as a pivotal CRE that mediates the induction of gene expression in response to salt stress [50]. To better understand the gene expression patterns of *BnaPAPs*, we conducted an analysis of the CREs within the promoter regions of 69 *PAP* genes (excluding 8 *BnaPAPs* due to incomplete or poor-quality promoter sequences) (Figure 6). The findings revealed that 49.28% (34/69) of the *BnaPAP* promoters contained P1BS elements, while 63.77% (44/69) harbored W-box elements and 20.29% (14/69) possessed the GT-1 element. Notably, genes with P1BS elements in their promoter regions are typically categorized as PSI genes (Figure 6, Table S6). For instance, *BnaPAP17s* exhibited enriched P1BS sites upstream of their promoters, with their transcripts being significantly upregulated in response to Pi stress. Similarly, three *BnaPAP12* genes had a P1BS site upstream, and their corresponding transcripts were also induced under low P conditions. However, *BnaA4.PAP12b* stood out as an exception, showing no notable increase in transcript accumulation under low P and lacking upstream P1BS sites. *BnaPAP24s* exhibit a dual responsiveness to both Pi deficiency and salt stress conditions, with their promoter sequences encompassing three distinct CRE: W-box, P1BS, and GT-1. We also identified several hormone and stress-responsive elements, including a GA-responsive element (GARE, TCTGTTG), MeJA-responsive elements (CGTCA-motif and TGACG-motif), an SA-responsive element (TCA-element, CCATCTTTTT), and a sulfur-responsive element (SURE, GAGAC) in the promoter of some *BnaPAP* genes. These findings suggest that *PAPs* may be regulated by diverse transcription factors in response to various environmental cues (Figure 6).

### 2.7. Key Regulatory Sequences Mediating Two BnaPAP10s Response to Pi Deficiency

**Figure 7:**
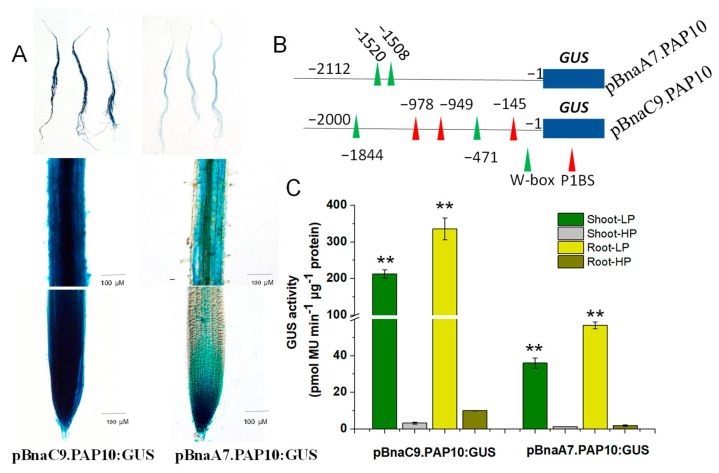
The activity and P-responsiveness of the *BnaC9.PAP10* and *BnaA7.PAP10* promoters. (**A**) GUS staining driven by the *BnaC9.PAP10* and *BnaA7.PAP10* promoter in the root. (**B**) A diagram showing the full-length of the *BnaC9.PAP10* and *BnaA7.PAP10* promoter. The locations of the P1BS and W-box element are indicated by a red and green triangle, respectively. (**C**) GUS enzymatic activity driven by different BnaPAP10 promoter fragments under LP (10 µM) and HP (250 µM) conditions. Asterisks indicate significant differences in GUS enzymatic activity compared with the treatment of high P (Student’s *t*-test, ** *p* < 0.01). Values are the means of three biological replicates with SE.

**Figure 8:**
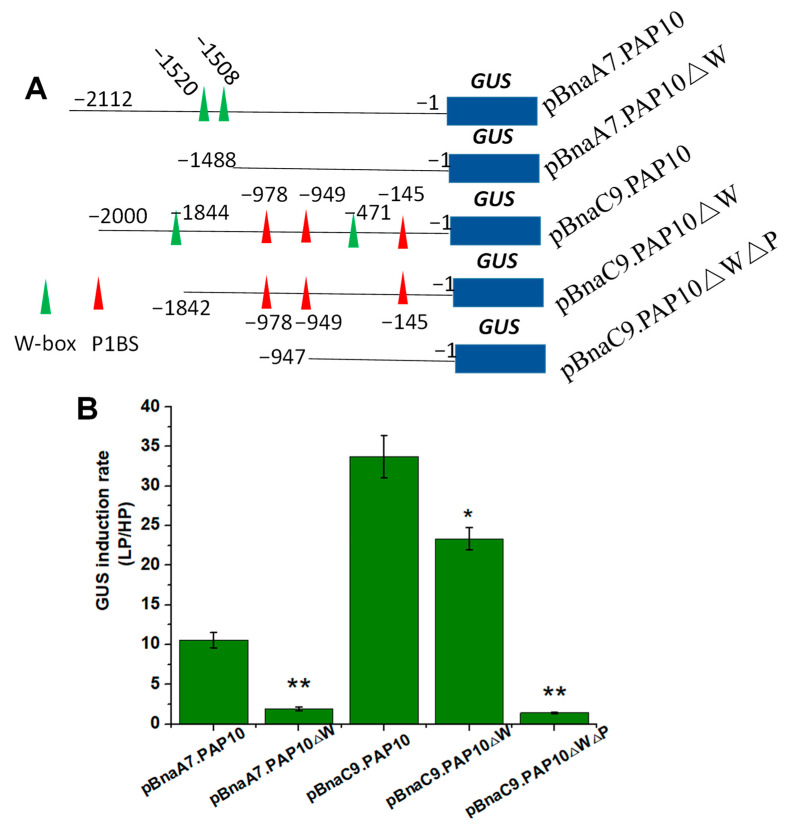
The deletion and mutation analysis of two *BnaPAP10* gene promoters. (**A**) A graphical depiction of various constructs, some with the targeted motifs removed. The P1BS and W-box motifs within the *BnaPAP10s* promoter are marked with red and green triangles, respectively. (**B**) The GUS induction rate was measured in the roots of transgenic *Arabidopsis* plants carrying these different constructs. The presented values represent the average of three biological replicates, with standard errors indicated. Asterisks indicate significant differences. * *p* < 0.05,** *p* < 0.01.

As previously mentioned, both *BnaC9.PAP10* and *BnaA7.PAP10* are genes induced by a Pi deficiency, yet *BnaC9.PAP10* demonstrates a robust induction, compared to the mild induction observed in *BnaA7.PAP10*. To delve deeper into the expression patterns of these two *BnaPAP10* genes and their crucial regulatory sequences, we cloned the full-length promoter fragments of *BnaA7.PAP10* (−2112 bp) and *BnaC9.PAP10* (−2000 bp), fusing them individually to the *GUS* reporter gene and transforming them into *Arabidopsis* plants. Representative T3-independent transgenic lines were cultivated in media under either high Pi (HP, 250 μM) or low Pi (LP, 10 μM) conditions for two weeks. Under HP conditions, the GUS activity driven by both *BnaPAP10* promoters was barely detectable in roots. However, upon exposure to LP conditions, a robust GUS staining was evident in roots of plants harboring the p*BnaC9.PAP10*:GUS construct. Conversely, plants carrying the p*BnaA7.PAP1*0:GUS construct displayed a weaker GUS staining, primarily confined to the vascular tissues of roots (Figure 7A). In a silico analysis of the promoter sequences revealed that *BnaC9.PAP10* harbors three copies of the P1BS motif and two copies of the W-box motif, both of which are associated with Pi signaling, whereas *BnaA7.PAP10* contains only two copies of the W-box element (Figure 6 and Figure 7B). The GUS enzyme activity assay results align with the staining outcomes, indicating a significantly elevated activity of *BnaC9.PAP10* relative to *BnaA7.PAP10* under a Pi-limited condition. To investigate the role of these W-box motifs in the Pi stress response of *BnaA7.PAP10*, we conducted targeted deletions of these motifs within the promoter fragment of p*BnaA7.PAP10*. The results revealed a significant decrease in the GUS induction rate in the plants with both W-box motifs simultaneously deleted (p*BnaA7.PAP10*ΔW) compared to the original p*BnaA7.PAP10* plants (Figure 8). These findings imply that, in addition to the P1BS element, the W-box also plays a pivotal role in regulating the Pi response, independently of the P1BS.

Since *BnaC9.PAP10* also contains two W-box motifs (−471 and −1844 upstream of the initiation codon), we constructed a vector with these two W-box motifs removed (p*BnaC9.PAP10*ΔW). While the removal of these W-box motifs led to a slight decrease in the GUS induction rate in p*BnaC9.PAP10*ΔW plants, compared to the original p*BnaC9.PAP10* plants, simultaneous elimination of both W-box motifs and the three P1BS motifs (p*BnaC9.PAP10*ΔWΔP) completely abolished the activity of *BnaC9.PAP10* (Figure 8B). This indicates that the P1BS motifs in the p*BnaC9.PAP10* promoter were the primary drivers of the Pi stress response and gene expression regulation. 

## 3. Discussion

In this study, a total of 77 *PAP* genes were identified in *B. napus*. The presence of ancient replication events coupled with a high retention rate in plant genomes results in a substantial number of duplicate genes. These duplicate genes facilitate the evolution of genes with novel functions [51]. *B. napus*, which is formed by the hybridization of the diploid *B. rapa* and *B. oleracea*, undergoes several rounds of whole-genome duplication (WGD), compared with *Arabidopsis* [41]. WGD-induced repeat regions typically involve duplications of all genes across a vast chromosomal area, rather than the duplication of individual or a limited number of genes. Consequently, these events generally contribute to the emergence of multicopy gene families in allotetraploid rapeseed. Our study uncovered that variations in homolog number among *PAPs* also exist in allotetraploid rapeseed. *Arabidopsis* possesses a single copy of each *PAP* gene [16]; however, the copy number of *PAPs* in *B. napus* ranges from one (*BnaPAP5*) to nine (*BnaPAP10s*), as shown in Table S2. It is noteworthy that *Brassica napus* possesses a higher number of *PAP* genes compared to other plant species, including rice, maize, soybean, and certain vegetable cultivars [25,26,27,28,29,30,31] (Figure S1). The presence of extra copies of *PAP* genes in *Brassica napus* offers potential for the evolution of supplementary functionalities, enhancing its adaptability to external environments [52].

Previous research has demonstrated that the *PAP* gene family plays pivotal roles in P acquisition and recycling within plants and is indispensable for the adaptation of plants to low-P environments [11]. Expression analysis indicated that 56% of *BnaPAP* genes showed a significant increase in expression under Pi deprivation conditions (Figure 4A), a higher percentage than that observed for *OsPAPs* (48%) [25] and *AtPAPs* (14%) [16]. Fourteen *BnaPAP* genes (*BnaA9.PAP1*, *BnaC8.PAP1*, *BnaC5.PAP2*, *BnaA6.PAP7*, *BnaA3.PAP10b*, *BnaA4.PAP12a*, *BnaC4.PAP12a*, *BnaA5.PAP15*, *4 BnaPAP17s* and *2 BnaPAP24s*) showed robust up-regulation in both roots and shoots in response to Pi deprivation (Figure 4A), which implied their potential involvement in plant P nutrition. It is interesting to note that *PAP* paralogs also exhibit diverse transcript profiles during Pi response. For instance, nine homologs of the *AtPAP10* gene exist in *Brassica napus;* however, only five *BnaPAP10s* respond to Pi deprivation indicating the functional differentiation of paralogs (Figure 4A). In contrast, 13 *BnaPAP* genes were constitutively expressed regardless of Pi availability, while 3 *BnaPAP* genes were downregulated under low Pi conditions. These findings indicate that, apart from their role in Pi acquisition, *PAPs* may possess other biochemical functions in plants.

Salt stress severely impairs plant growth and development, primarily by disrupting ionic balance and water relations within their cells [43]. Several studies have indicated that *PAP* also plays roles in response to salt stress. For instance, *GmPAP3*, a unique purple acid phosphatase-like gene in soybean, is induced by NaCl stress (rather than by a Pi deficiency) and helps alleviate oxidative damage caused by salinity and osmotic stresses. *AtPAP17* and *AtPAP26* are not only responsive to Pi deficiency but are also involved in salt tolerance [20,21,22,35,36]. However, the precise transcriptional regulation of *PAP* genes in response to salt stress in *Brassica napus* remains elusive. In this study, we conducted a comprehensive investigation of the expression profiles *BnaPAPs* in both leaves and roots under NaCl stress. Our analysis of RNA-seq data [53,54] revealed that 17 *BnaPAPs* were induced specifically in the roots, while 12 were induced specifically in the shoots. *BnaA1.PAP24* was induced both in the shoots and roots (Figure 4B). Certain *BnaPAP* genes, exemplified by *BnaA5.PAP15* and *BnaPAP24s*, demonstrate the responsiveness to both Pi deficiency and salt stress. This dual responsiveness indicates their potential involvement in complex stress-signaling pathways and adaptive mechanisms in plants.

Through a comprehensive analysis of expression levels and GUS activity, we have confirmed that *BnaC9.PAP10* and *BnaA7.PAP10* genes both respond to Pi deficiency, but exhibit significant differential fold changes in their induction under Pi stress conditions. (Figure 5 and Figure 7A,C). There are many factors that regulate gene expression, such as methylation, acetylation and promoter activity [55]. The CREs in the promoters of stress-responsive genes aid in elucidating their regulatory mechanisms. An in silico analysis of the promoter fragment revealed that *BnaC9.PAP10* harbors three P1BS motifs and two W-box motifs, both of which are linked to Pi signaling. In contrast, *BnaA7.PAP10* only possesses two copies of the W-box elements. The targeted deletion of the two W-box motifs in *BnaA7.PAP10* led to a significant decrease in GUS induction, indicating their crucial role in up-regulating *BnaA7.PAP10* in response to Pi starvation. The truncations and deletions analysis of *BnaC9.PAP10* promoter suggested that P1BS and W-box elements enhance its up-regulation under Pi stress. Transgenic plants with targeted deletions of P1BS motifs lost most of their Pi responsiveness (Figure 8), highlighting that the P1BS in the p*BnaC9.PAP10* promoter is the primary motif responsible for responding to Pi stress and regulating gene expression. Given previous research suggesting that not all P1BS motifs in a promoter respond equally to Pi starvation [56], future studies should investigate the impact of P1BS number and position on gene expression activation. Additionally, considering the complexity of Pi signaling regulation in plants, we cannot exclude the possibility that other regulatory elements within the promoters of the two *BnaPAP10* paralogs may also mediate their Pi stress response. Furthermore, epigenetic regulation could potentially influence the expression of the two *PAP* paralogs [57]. In summary, our study revealed CRE variations in the promoters of the two *BnaPAP10* paralogs, resulting in differential expression in response to Pi stress.

## 4. Materials and Methods

### 4.1. Identification of PAP Genes in B. napus and Motif Analysis

Using the BLASTP search program within the BRAD database ^1^ [58], *PAP* genes were identified in *B. napus* based on a comparison with 29 PAP protein sequences from *Arabidopsis* [16]. All retrieved PAP protein sequences aligned using ClustalW program to identify the five conserved blocks of amino acids (GDXG/GDXXY/GNH(D/E)/VXXH/GHXH). The presence of metallophospho-associated proteins was confirmed by searching all extracted PAP-like protein sequences against the Pfam database ^2^ [59] and the SMART database ^3^ [60], utilizing their default parameters.

### 4.2. Multiple Sequence Alignment and Phylogenetic Analysis

We utilized ClustalW [61], integrated within MEGA (Molecular Evolutionary Genetics Analysis) version 7.0 ^4^ [62], to align the full-length protein sequences of PAPs. Following these alignments, phylogenetic trees were constructed using the neighbor-joining (NJ) method [63]. The necessary parameters for the tree construction included the application of Poisson correction for distance estimation, pairwise deletion to address missing data, and bootstrapping with 1000 replicates using random seeds to ensure the statistical robustness of the phylogenetic trees.

### 4.3. Gene Structure, Protein Properties and Promoter Elements Analysis of BnaPAPs

We inspected the exon-intron organizations of *BnaPAP* family genes using the online gene structure display server ^5^, relying on alignments of full-length coding sequences (CDS) with their corresponding genomic sequences [64]. To uncover the molecular attributes of BnaPAPs, we utilized the ExPASy ProtoParam tool ^6^ to ascertain the amino acid (AA) count and composition, molecular weight (MW), isoelectric point (pI), grand average of hydropathicity (GRAVY), and instability index (IIs) [65]. For predicting the occurrence and position of signal peptide cleavage sites within the AA sequences of BnaPAPs, we employed the SignalP v. 4.1 online tool ^7^ [66]. The subcellular localization of BnaPAP proteins was analyzed using the TargetP1.1 server ^8^ with default settings [67]. To predict N-glycosylation sites in BnaPAP proteins, we leveraged the NetNGlyc 1.0 application [68]. For identifying transmembrane helices in BnaPAPs, their AA sequences were subjected to the TMHMM v. 2.0 program ^9^. The Plant CARE web signal scan ^10^ [69] was used to search for *cis*-acting elements within the 2 kb upstream region of the start codon of the *BnaPAPs* gene.

### 4.4. Plant Materials and Treatments

In this study, the rapeseed cultivar named “eyouchangjia” was utilized. The seedlings were cultivated in a greenhouse, where they were nurtured in hoagland solution under controlled conditions: a 16 h daylight period at 24 °C, followed by an 8 h night period at 22 °C, maintained at a relative humidity of 60–70%. Previous research showed an RNA-seq experiment where hydroponically grown seedlings were exposed to 0 µM or 250 µM Pi for 10 days before harvesting leaves and roots for RNA extraction [53]. To investigate gene expression during Pi starvation and resupply, seedlings were treated with P-free nutrient solution (0 µM Pi), and samples were collected at 0, 1 d, 3 d, and 7 d. Then, plants were returned to Pi-sufficient solution (250 µM Pi), and leaves and roots were harvested 1 d later. Additionally, a pot culture experiment was conducted to analyze the *BnaPAPs* expression in different tissues under low (5 mg P_2_O_5_ kg^−1^ soil, LP) and high (150 mg P_2_O_5_ kg^−1^ soil, HP) Pi conditions. RNA was extracted from old and young leaves, flowers, pods, and pod peduncles of 6-month-old plants. For salt stress treatment, 7-day-old *B. napus* seedlings were hydroponically grown in NaCl-free solution for 10 days, then exposed to 200 mM NaCl for 1 day before sampling. All experiments incorporated three biological replicates, and the samples were promptly frozen in liquid nitrogen and kept at −80 °C until RNA extraction was performed.

### 4.5. BnaPAPs Expression Patterns Under Pi Starvation: RNA-Seq Analysis

Utilizing the RNA sequencing data, outlined in Table S5, from our research team’s publication [53,54], we analyzed the expression patterns of *BnaPAP* genes under two P conditions: high-phosphate (HP, with 250 µM Pi) and low-phosphate (LP, with 0 µM Pi). For salt stress treatment, 7-day-old *B. napus* seedlings were hydroponically grown in NaCl-free solution for 10 days, then exposed to 200 mM NaCl for 1 d before sampling. We processed FPKM or TPM values and subsequently generated a heatmap with the assistance of TBtools software V2.142 [70].

### 4.6. Expression Analysis by qRT-PCR

Plant samples were processed for total RNA extraction using TRIzol reagent, following the manufacturer’s guidelines provided by Invitrogen (USA). The synthesis of first-strand cDNA was carried out using M-MLV reverse transcriptase and oligo(dT), adhering to the protocol supplied by Promega (USA). Quantitative real-time RT-PCR reactions were conducted on the CFX96™ Real-Time PCR Detection System (Bio-Rad, USA), employing the SYBR Green system sourced from Toyobo (Japan). The PCR amplification protocol involved an initial denaturation at 95 °C for 2 min, followed by 40 cycles of denaturation at 95 °C for 30 s, annealing at 60 °C for 30 s, and extension at 72 °C for 1 min. The relative expression levels of *BnaPAP* genes were determined using the 2^−∆∆CT^ method [71], with *Actin* serving as the reference gene [72]. The specific primers utilized for qRT-PCR analysis are detailed in Table S7.

### 4.7. Vector Construction and Genetic Transformation

To create chimeric genes where the GUS coding sequence is controlled by various lengths of the *BnaA7.PAP10* and *BnaC9.PAP10* promoters, we obtained a series of promoter fragments via PCR or fusion PCR. This was conducted using genomic DNA from the rapeseed variety “eyouchangjia” as the template, along with specific primers listed in Table S7. The resulting PCR products were then inserted into the binary vector DX2181b, generating a total of five plasmids. After confirming their sequences, all constructs were introduced into the *A. tumefaciens* strain GV3101. These constructs were subsequently transformed into *Arabidopsis thaliana* using the floral-dip method [73]. The T3 progeny of the hygromycin-resistant transformants were subjected to GUS staining under various Pi treatments.

### 4.8. GUS Activity Analysis in Transgenic Arabidopsis

For GUS staining, the leaves were infiltrated with staining solution (Real Times, Beijing, China) for 12 h and decolored in 75% ethanol. The GUS activity was quantitatively determined using the substrate 4-methylumbelliferyl-b-D-glucuronide (4-MUG), and the reaction product 4-methylymbelliferone (4-MU) was detected using TECAN Infinite M200. GUS activity was shown in units of nmol 4-methylumbel-liferone produced per min per microgram of protein.

### 4.9. Statistical Analysis

For statistical analyses, SPSS was used. Data were presented as the means ± SD based on at least three independent replicates. Statistically significant differences among the treatments for multiple comparisons were performed using Turkey’s test of one-way analysis of variance (ANOVA), whereas differences between pairs of treatments were conducted using Student’s *t*-test (with an overall significance level of *p* = 0.05).

## 5. Conclusions

In this study, we discovered a total of 77 full-length *PAP* homologs in the allotetraploid rapeseed genome (AnAnCnCn). We conducted a comprehensive analysis of these *BnaPAP* genes, including the examination of conserved metallophos motifs, signal peptides, phylogenetic relationships, physio-chemical properties, CREs, and responses to Pi deficiency and NaCl stress of all identified rapeseed *PAP* genes. Additionally, we performed a deletion analysis to pinpoint the motifs responsible for the differential expression of two PSI-responsive *BnaPAP10* genes, namely *BnaC9.PAP10* and *BnaA7.PAP10*, under Pi stress conditions. Overall, our findings describe a comprehensive view of the *BnaPAP* family and provide an integrated insight into their family evolution and the candidates for increasing P use efficiency and salt tolerance.

## Data Availability

Data are contained within the article and supplementary materials.

## Supplementary Materials

The following supporting information can be downloaded at: https://www.mdpi.com/article/10.3390/plants14030461/s1, Figure S1: Homologous gene number of the *PAP* family members in plant species.; Figure S2: Gene structure diagram of *BnaPAP* genes; Figure S3: Expression profiles of 12 selected *PAP* family genes in the different tissues of *Brassica napus* under Pi-deficient and Pi-sufficient conditions. Table S1: Conserved residues and domains for identified putative *BnaPAPs*; Table S2: Molecular characterization of the *PAP* genes in *Brassica napus* and their subcellular location.; Table S3: The tandemly duplicated genes detected in the *PAP* family genes in *Brassica napus*.; Table S4. Normalized FPKM of detected *BnaPAPs* in RNA-seq analysis.; Table S5: Transcripts Per Million (TPM) data of detected *BnaPAPs* in RNA-seq analysis.; Table S6: Number and position of *cis*-regulatory elements related to Pi stress present in the promoters of *PAP* genes in *B.napus.;* Table S7: Primers used in the present study.

## Abbreviation

At	*Arabidopsis thaliana*
Bna	*Brasssica napus*
BRAD	Brassica Database
CDS	Coding sequence
CRE	*cis*-acting regulatory element
MEME	Multiple expectation maximization for motif elicitation
MW	Molecular weight
qRT-PCR	quantitative reverse-transcription polymerase chain reaction
TAIR	The *Arabidopsis* Information Resource
TF	transcription factor
^1^	BRAD (http://brassicadb.cn/#/BLAST/, accessed on 17 March 2023)
^2^	Pfam database (http://pfam.xfam.org/, accessed on 17 March 2023)
^3^	SMART database (http://smart.embl-heidelberg.de/, accessed on 17 March 2023)
^4^	MEGA v7 (http://www.megasoftware.net/, accessed on 20 March 2023)
^5^	Gene Structure Display Server (http://gsds.cbi.pku.edu.cn/, accessed on 27 March 2023)
^6^	ExPASy ProtoParam (http://www.expasy.org/tools/protparam.html, accessed on 20 March 2023)
^7^	SignalP v. 4.1 (http://www.cbs.dtu.dk/services/SignalP/, accessed on 17 March 2023)
^8^	TargetP1.1 server (http://www.cbs.dtu.dk/services/TargetP/, accessed on 17 March 2023)
^9^	TMHMM v. 2.0 (http://www.cbs.dtu.dk/services/TMHMM/, accessed on 17 March 2023)
^10^	Plant CARE (http://bioinformatics.psb.ugent.be/webtools/plantcare/html/, accessed on 17 March 2023)
