# Supplementary material for: Identification and Expression Analysis of Acid Phosphatase Gene (PAP) in Brassica napus: Effects of cis-Acting Elements on Two BnaPAP10 Genes in Response to Phosphorus Stress
Source: Plants (Basel). 2025 Feb 5;14(3):461. doi: 10.3390/plants14030461 (PMC11819708; doi:10.3390/plants14030461)
Supplement: Supplementary file 1 [file plants-14-00461-s001.zip › Supplemental figures.pdf]

Plant Species	PAP Gene number	Genome size（Mb）	Average number of PAP family per Mb
Arabidopsis thaliana	29	125	0.2320
Brassica rapa	37	485	0.0763
Brassica oleracea	44	630	0.0698
Brassica napus	77	1345	0.0572
Glycine max	35	1100	0.0318
Solanum lycopersicum	27	950	0.0284
Cucumis sativus	18	350	0.0514
Cucumis melo	15	450	0.0333
Citrullus lanatus	16	425	0.0376
Solanum pennellii	28	942	0.0297
Solanum tuberosum	22	844	0.0261
Solanum melongena	19	1093	0.0174
Capsicum annuum	22	3480	0.0063
Jatropha curcas	25	410	0.0610
Cicer arietnum	25	738	0.0339
Zea mays	33	2300	0.0143
Oryza sativa	26	466	0.0558

Figure S1. Homologous gene number of the *PAP* family members in plant species.

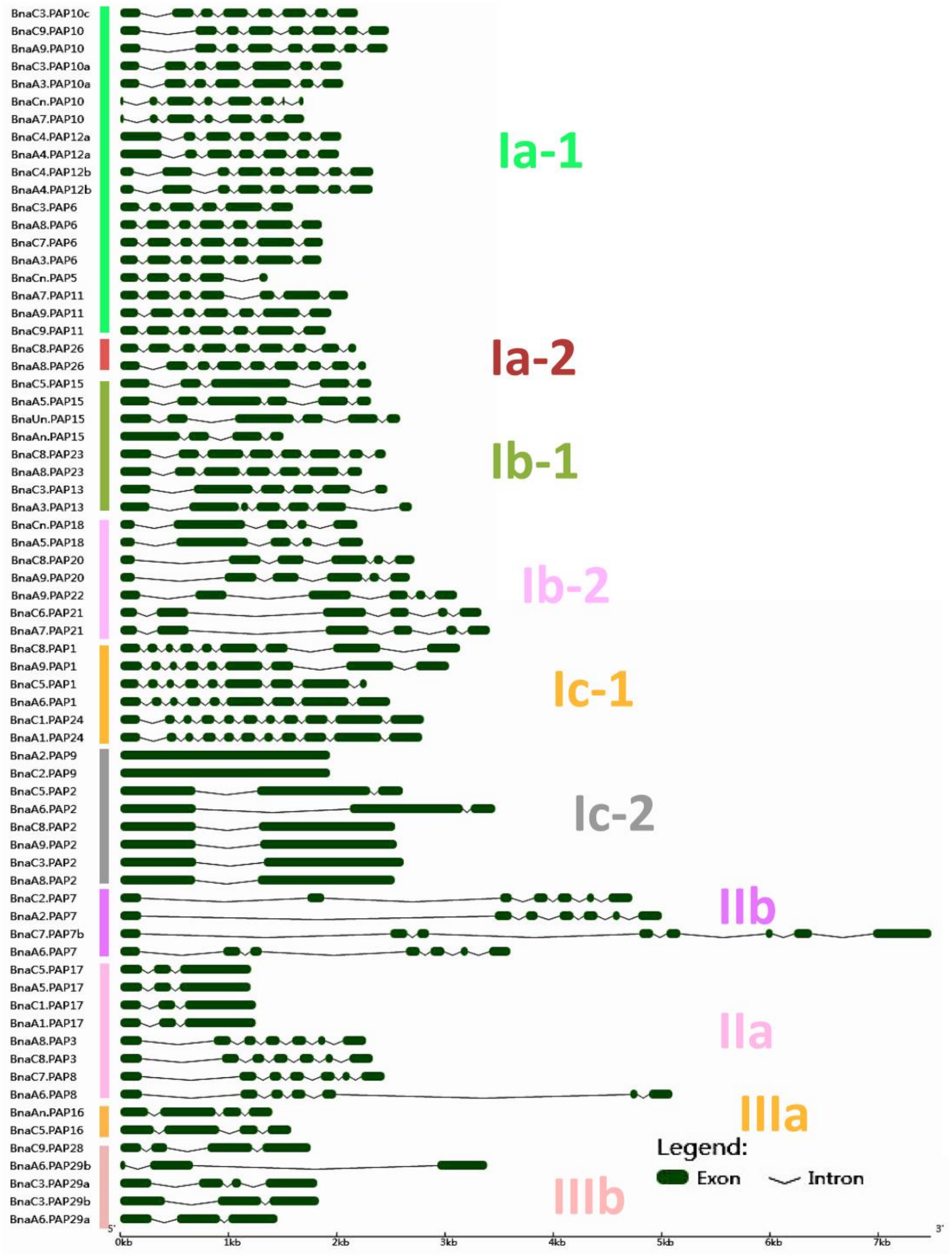

Figure S2. Gene structure diagram of *BnaPAP* genes. Exons were represented by green boxes and introns by black lines. The sizes of exons and introns could be estimated using the scale at bottom.

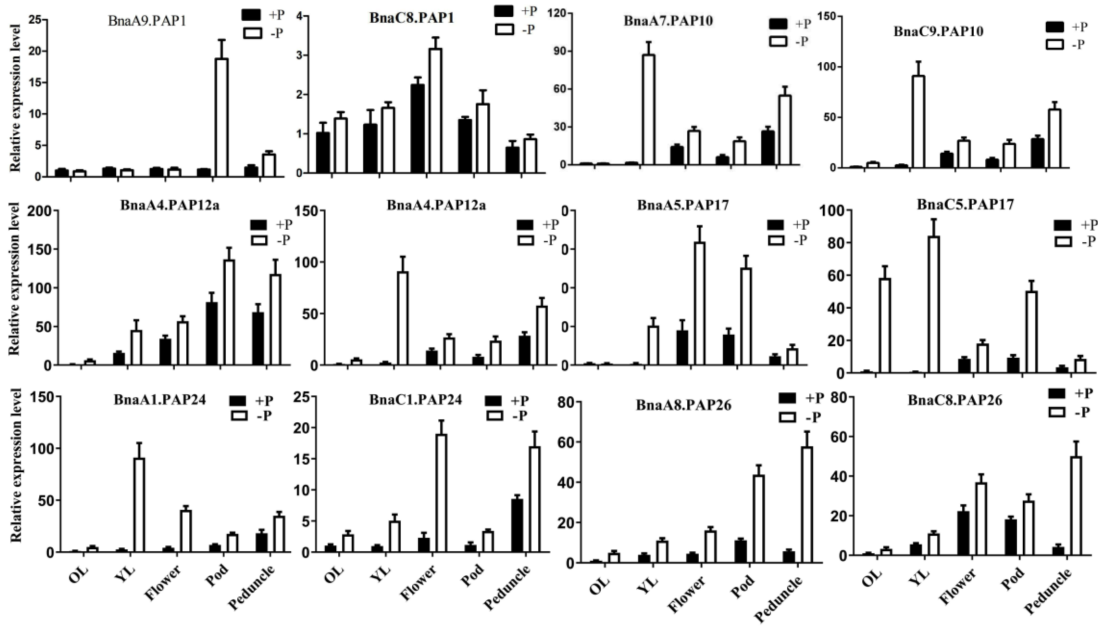

Figure S3. Expression profiles of 12 selected *PAP* family genes in the different tissues of *Brassica napus* under Pi-deficient and Pi-sufficient conditions. The relative expression of *BnaPAPs* was analyzed by qRT-PCR in old leaves (OL), young leaves (YL), flowers, pods, and pod peduncles. The expression level of *BnaPAPs* in old leaves under P-sufficient condition was set as 1. Values represent the mean ± SD of three biological replicates.
