# Supplementary material for: Identification and Expression Analysis of Acid Phosphatase Gene (PAP) in Brassica napus: Effects of cis-Acting Elements on Two BnaPAP10 Genes in Response to Phosphorus Stress
Source: Plants (Basel). 2025 Feb 5;14(3):461. doi: 10.3390/plants14030461 (PMC11819708; doi:10.3390/plants14030461)
Supplement: Supplementary file 1 [file plants-14-00461-s001.zip › Supplemental Tables.pdf]

Table S1 Conserved residues and domains for identified putative BnaPAPs

Gene ID	Proposed Name	PAP Conserved Residues					Conserved Domain[a]
		GDXG	GDXXY	GNH(D/E)	VXXH	GHXH	
**BnaC08g40180D**	*BnaC8.PAP1*	GDMG	KDLKK	GNHE	FLAH	GHAH	MPP_PAPs, fn3_PAP superfamily, Pur_ac_phosph_N
**BnaC05g10090D**	*BnaC5.PAP1*	GDMG	KDLNK	GNHE	FLAH	GHAH	MPP_PAPs, fn3_PAP superfamily, Pur_ac_phosph_N
**BnaA09g46120D**	*BnaA9.PAP1*	GDMG	KDLKK	GNHE	FLAH	GHAH	MPP_PAPs, Pur_ac_phosph_N, fn3_PAP
**BnaA06g08770D**	*BnaA6.PAP1*	GDMG	KDLNK	GNHE	FLAH	GHAH	MPP_PAPs, fn3_PAP superfamily, Pur_ac_phosph_N
**BnaC08g40040D**	*BnaC8.PAP2*	GDMG	RDIEN	GNHE	VQGH	GHVH	MPP_PAPs, fn3_PAP superfamily, Pur_ac_phosph_N
**BnaC05g10180D**	*BnaC5.PAP2*	GDMG	RDIEN	GNHE	VQGH	GHVH	MPP_PAPs, fn3_PAP superfamily, Pur_ac_phosph_N
**BnaC03g56550D**	*BnaC3.PAP2*	GDMG	RDIEN	GNHE	VQGH	GHVH	MPP_PAPs, fn3_PAP superfamily, Pur_ac_phosph_N
**BnaA09g45970D**	*BnaA9.PAP2*	GDMG	RDIEN	GNHE	VQGH	GHVH	MPP_PAPs, Pur_ac_phosph_N, fn3_PAP
**BnaA08g24200D**	*BnaA8.PAP2*	GDMG	RDIEN	GNHE	VQGH	GHVH	MPP_PAPs, Pur_ac_phosph_N, fn3_PAP
**BnaA06g08860D**	*BnaA6.PAP2*	GDMG	RDIEN	GNHE	VQGH	GHVH	MPP_PAPs, Pur_ac_phosph_N, fn3_PAP
**BnaA08g31430D**	BnaA8.PAP3	GLDD	GNHDY	EIVE	VVGH	GHDH	MPP_ACP5
**BnaC08g16330D**	BnaC8.PAP3	GLDD	GNHDY	EIVE	VVGH	GHDH	MPP_ACP5
**BnaCnng74060D**	*BnaCn.PAP5*	GDLG	GDLSY	GNHE	None	None	PLN02533 superfamily
**BnaC07g51090D**	*BnaC7.PAP6*	GDLG	GDLSY	GNHE	VIVH	GHVH	MPP_PAPs, Pur_ac_phosph_N
**BnaA03g59200D**	*BnaA3.PAP6*	GDLG	GDLSY	GNHE	VIVH	GHVH	MPP_PAPs, Pur_ac_phosph_N
**BnaC03g61960D**	*BnaC3.PAP6*	GDLG	GDLNY	GNHE	VMVH	GHVH	MPP_PAPs, Pur_ac_phosph_N
**BnaA08g15210D**	*BnaA8.PAP6*	GDLG	GDLNY	GNHE	VMVH	GHVH	MPP_PAPs, Pur_ac_phosph_N
**BnaC07g21320D**	*BnaC7.PAP7a*	GDTD	GNHDY	GMVE	VVGH	GHDH	MPP_ACP5
**BnaC07g21290D**	*BnaC7.PAP7b*	GDTD	GNHDY	GMVE	VVGH	GHDH	MPP_ACP5
**BnaC02g34120D**	*BnaC2.PAP7*	GDTD	GNHDY	GMVE	VVGH	GHDH	MPP_ACP5
**BnaA06g34460D**	*BnaA6.PAP7*	GDTD	GNHDY	GMVE	VVGH	GHDH	MPP_ACP5
**BnaA02g26060D**	*BnaA2.PAP7*	GDTD	GNHDY	GMVE	VVGH	GHDH	MPP_ACP5

**BnaC07g21340D**	*BnaC7.PAP8*	SPYD	GNHDY	DIVD	VVGH	GHDH	MPP_ACP5
**BnaA06g34450D**	*BnaA6.PAP8*	SPYD	GNHDY	DIVD	VVGH	GHDH	MPP_ACP5
**BnaA02g36950D**	*BnaA2.PAP9*	GDMG	RDIKS	GNHE	VQGH	GHVH	MPP_PAPs, fn3_PAP superfamily, Pur_ac_phosph_N
**BnaC02g35040D**	*BnaC2.PAP9*	GDMG	RDIKS	GNHE	VQGH	GHVH	MPP_PAPs, fn3_PAP superfamily, Pur_ac_phosph_N
**BnaCnng72040D**	*BnaCn.PAP10*	GDLG	GDLSY	GNHE	VLNH	GHVH	MPP_PAPs
**BnaC09g08970D**	*BnaC9.PAP10*	RDLG	GDFSY	GNHE	VLMH	GHVH	MPP_PAPs, Pur_ac_phosph_N
**BnaC03g46400D**	*BnaC3.PAP10a*	GDLG	GDLSY	GNHE	VLNH	GHVH	MPP_PAPs, Pur_ac_phosph_N
**BnaC03g46310D**	*BnaC3.PAP10b*	GDLG	GDFSY	GNHE	VLMH	GHVH	MPP_PAPs, Pur_ac_phosph_N
**BnaC03g46270D**	*BnaC3.PAP10c*	GDLG	GDFSY	GNHE	VLMH	GHVH	MPP_PAPs, Pur_ac_phosph_N
**BnaA09g08700D**	*BnaA9.PAP10*	GDLG	GDFSY	GNHE	VLMH	GHVH	MPP_PAPs, Pur_ac_phosph_N
**BnaA07g15630D**	*BnaA7.PAP10*	RDLG	GDLSY	GNHE	VLNH	GHVH	MPP_PAPs
**BnaA03g39100D**	*BnaA3.PAP10a*	GDLG	GDLSY	GNHE	VLNH	GHVH	MPP_PAPs, Pur_ac_phosph_N
**BnaA03g39080D**	*BnaA3.PAP10b*	GDLG	GDFSY	GNHE	VLMH	GHVH	MPP_PAPs, Pur_ac_phosph_N
**BnaA09g09490D**	*BnaA9.PAP11*	GDLG	GDLSY	GNHE	VLVH	GHVH	MPP_PAPs, Pur_ac_phosph_N
**BnaA07g01870D**	*BnaA7.PAP11*	GDLG	GDLSY	GNHE	VLVH	GHVH	MPP_PAPs, Pur_ac_phosph_N
**BnaC09g09650D**	*BnaC9.PAP11*	GDLG	GDLSY	GNHE	VLVH	GHVH	MPP_PAPs, Pur_ac_phosph_N
**BnaC04g38420D**	*BnaC4.PAP12a*	GDLG	GDLSY	GNHE	VLVH	GHVH	MPP_PAPs, Pur_ac_phosph_N
**BnaC04g38390D**	*BnaC4.PAP12b*	GDLG	GDLSY	GNHE	VLVH	GHVH	MPP_PAPs, Pur_ac_phosph_N
**BnaA04g15430D**	*BnaA4.PAP12a*	GDLG	GDLSY	GNHE	VLVH	GHVH	MPP_PAPs, Pur_ac_phosph_N
**BnaA04g15420D**	*BnaA4.PAP12b*	GDLG	GDLSY	GNHE	VLVH	GHVH	MPP_PAPs, Pur_ac_phosph_N
**BnaC03g18120D**	*BnaC3.PAP13*	GDLG	GDFSY	GEHE	None	None	MPP_PAPs, Pur_ac_phosph_N
**BnaA03g15100D**	*BnaA3.PAP13*	GDLG	GDFSY	GEHE	None	None	MPP_PAPs, Pur_ac_phosph_N
**BnaA05g01020D**	*BnaA5.PAP14*	NHDG	None	GDYV	None	GHDH	MPP_Dcr2
**BnaUnng02540D**	*BnaUn.PAP15*	GDTG	GDVSY	GNHE	VTWH	GHVH	MPP_PAPs, Pur_ac_phosph_N
**BnaAnng39640D**	*BnaAn.PAP15*	GDTG	GDVSY	GNHE	VTWH	GHVH	MPP_PAPs, Pur_ac_phosph_N
**BnaC05g44840D**	*BnaC5.PAP15*	GDLG	GDVSY	GNHE	VSWH	GHVH	MPP_PAPs, Pur_ac_phosph_N
**BnaA05g30450D**	*BnaA5.PAP15*	GDLG	GDVSY	GNHE	VSWH	GHVH	MPP_PAPs, Pur_ac_phosph_N
**BnaAnng16610D**	*BnaAn.PAP16*	NHDD	GDVSY	ESSD	None	GHNH	MPP_Dcr2
**BnaC05g42920D**	*BnaC5.PAP16*	NHDD	GDVSY	ESSD	None	GHNH	MPP_Dcr2
**BnaC05g35720D**	*BnaC5.PAP17*	SEYD	GNHDY	ELVE	VVGH	GHDH	MPP_ACP5
**BnaC01g34650D**	*BnaC1.PAP17*	SEHD	GNHDY	ELVE	VVGH	GHDH	MPP_ACP5

**BnaA05g22460D**	*BnaA5.PAP17*	SEYD	GNHDY	ELVE	VVGH	GHDH	MPP_ACP5
**BnaA01g27810D**	*BnaA1.PAP17*	SEHD	GNHDY	ELVE	VVGH	GHDH	MPP_ACP5
**BnaCnng46140D**	*BnaCn.PAP18*	GDLG	GDLSY	GNHE	ALFH	GHVH	PLN02533 superfamily
**BnaA05g20440D**	*BnaA5.PAP18*	GDLG	GDLSY	GNHE	ALFH	GHVH	PLN02533 superfamily
**BnaC08g23980D**	*BnaC8.PAP20*	GDLG	GDLSY	GNHE	AVIH	GHVH	PLN02533
**BnaA09g33190D**	*BnaA9.PAP20*	GDLG	GDLSY	GNHE	AVIH	GHVH	PLN02533
**BnaC06g14110D**	*BnaC6.PAP21*	GDLG	GDLSY	GNHE	AVMH	GHIH	PLN02533 superfamily
**BnaA07g15780D**	*BnaA7.PAP21*	GDLG	GDLSY	GNHE	AVMH	GHIH	PLN02533 superfamily
**BnaA09g33200D**	*BnaA9.PAP22*	GDLG	GDLSY	GNHE	VLLH	GHVH	PLN02533 superfamily
**BnaC08g08870D**	*BnaC8.PAP23*	GDLG	GDLTY	GNHE	ATMH	GHVH	PLN02533 superfamily
**BnaA08g05220D**	*BnaA8.PAP23*	GDLG	GDLTY	GNHE	ATMH	GHVH	PLN02533 superfamily
**BnaC01g16730D**	*BnaC1.PAP24*	GDMG	KDLKN	GNHE	FIAH	GHVH	MPP_PAPs, fn3_PAP superfamily, Pur_ac_phosph_N
**BnaA01g14240D**	*BnaA1.PAP24*	GDMG	KDLKN	GNHE	FIAH	GHVH	MPP_PAPs, fn3_PAP superfamily, Pur_ac_phosph_N
**BnaC08g08080D**	*BnaC8.PAP26*	GDLG	GDLSY	GNHE	VLMH	GHVH	PLN02533 superfamily
**BnaA08g07270D**	*BnaA8.PAP26*	GDLG	GDLSY	GNHE	VLMH	GHVH	PLN02533 superfamily
**BnaC09g53400D**	*BnaC9.PAP28*	GNHD	None	GNRE	None	GHDH	MPP_Dcr2
**BnaC02g11790D**	*BnaC2.PAP28*	GNHD	None	GNRE	None	GHDH	MPP_Dcr2
**BnaA10g11170D**	*BnaA10.PAP28*	GNHD	None	GNRE	None	GHDH	MPP_Dcr2
**BnaCnng15730D**	*BnaCn.PAP29*	GNHD	None	GNYS	None	GHDH	MPP_Dcr2
**BnaC03g51100D**	*BnaC3.PAP29a*	GNHD	None	GNYS	None	GHDH	MPP_Dcr2
**BnaC03g51090D**	*BnaC3.PAP29b*	GNHD	None	GNYS	None	GHDH	MPP_Dcr2, SbcD superfamily
**BnaA09g06490D**	*BnaA9.PAP29*	GNHD	None	GNYS	None	GHDH	MPP_Dcr2
**BnaA06g22560D**	*BnaA6.PAP29a*	GNHD	None	GNYS	None	GHDH	MPP_Dcr2, SbcD superfamily
**BnaA06g22550D**	*BnaA6.PAP29b*	GNHD	None	GNYS	None	GHDH	MPP_Dcr2, SbcD superfamily

Table S2. Molecular characterization of the *PAP* genes in *Brassica napus* and their subcellular location.

Gene ID	Gene name	NO. of exon	Protein properties		signal peptide (length, cleave site)	number of N-glycosylation site	N-terminal transmembrane helix (length, location)	TargetP	Best hit
			Amino acid	MW (KDa)					
**BnaC08g40180D**	*BnaC8.PAP1*	9	615	68.5	27, VES-HK	4	20, 7-26	SP	*AtPAP1* *AT1G13750* (A Block)
**BnaC05g10090D**	*BnaC5.PAP1*	9	532	59.9	22, VNC-HI.	3	No	SP	
**BnaA09g46120D**	*BnaA9.PAP1*	9	621	69.3	24, ALG-GR	4	18, 7-24	SP	
**BnaA06g08770D**	*BnaA6.PAP1*	9	619	69.4	22, GGA-IQ	2	20, 7-26	SP	
**BnaC08g40040D**	*BnaC8.PAP2*	2	651	73.1	21, ANA-KA	5	20, 607-629	SP	*AtPAP2* *AT1G13900* (A Block)
**BnaC05g10180D**	*BnaC5.PAP2*	3	655	73.5	21, ANA-EA	2	20, 618-637	SP	
**BnaC03g56550D**	*BnaC3.PAP2*	2	663	74.5	23, ANA-KA	4	23, 611-633	SP	
**BnaA09g45970D**	*BnaA9.PAP2*	2	652	73.4	21, ANA-KA	3	23, 607-629	SP	
**BnaA08g24200D**	*BnaA8.PAP2*	2	651	73.1	20, ANA-KA	6	23, 609-631	SP	
**BnaA06g08860D**	*BnaA6.PAP2*	3	655	73.5	22, ANA-EA	3	20, 618-637	SP	
**BnaA08g31430D**	*BnaA8.PAP3*	7	334	38	29, STA-EL	1	18, 12-29	SP	*AtPAP3* *AT1G14700* (A Block)
**BnaC08g16330D**	*BnaC8.PAP3*	7	334	38.1	29, STA-EL	1	18, 12-29	SP	
**BnaCnng74060D**	*BnaCn.PAP5*	5	265	29.5	24 ,SHA-GV	1	No	SP	*AtPAP5* AT1G52940 (C Block)
**BnaC07g51090D**	*BnaC7.PAP6*	7	460	52.2	22, ING-GM	3	No	SP	*AtPAP6* *AT1G56360*
**BnaA03g59200D**	*BnaA3.PAP6*	7	460	52.2	22, ING-GM	3	No	SP	

**BnaC03g61960D**	*BnaC3.PAP6*	6	400	46.1	Other	1	No	other	
**BnaA08g15210D**	*BnaA8.PAP6*	7	459	52.4	19, ING-GI	1	No	SP	
**BnaC07g21320D**	*BnaC7.PAP7a*	2	319	36.7	Other	1	No	other	
**BnaC07g21290D**	*BnaC7.PAP7b*	8	491	55.9	24, SLS-KL	1	23, 428-450	SP	*AtPAP7* *AT2G01880* (K Block)
**BnaC02g34120D**	*BnaC2.PAP7*	7	332	38	26, SFS-KL	2	20, 7-26	SP	
**BnaA06g34460D**	*BnaA6.PAP7*	7	320	36.6	22, SLS-KL	2	No	SP	
**BnaA02g26060D**	*BnaA2.PAP7*	7	332	37.9	26, SFS-KL	2	20, 7-26	SP	
**BnaC07g21340D**	*BnaC7.PAP8*	7	334	38.2	29, SRA-EL	1	No	SP	*AtPAP8* AT2G01890 (K Block)
**BnaA06g34450D**	*BnaA6.PAP8*	7	334	38.2	29, STA-EL	2	No	SP	
**BnaA02g36950D**	*BnaA2.PAP9*	1	644	73.2	20, VHS-TL	3	23, 2-24; 597 -619	SP	*AtPAP9* *AT2G03450* (K Block)
**BnaC02g35040D**	*BnaC2.PAP9*	1	644	73.1	18, VHS-TP	3	23, 597-619	SP	
**BnaCnng72040D**	*BnaCn.PAP10*	9	283	32.7	Other	5	No	mTP	*AtPAP10* *AT2G16430* (H Block)
**BnaC09g08970D**	*BnaC9.PAP10*	1	467	54	28, CHG-GR	2	23, 7-29	SP	
**BnaC03g46400D**	*BnaC3.PAP10a*	7	466	53.7	25, CNG-GI	4	No	SP	
**BnaC03g46310D**	*BnaC3.PAP10b*	8	460	53.1	22, CHG-GT	2	23, 7-29	SP	
**BnaC03g46270D**	*BnaC3.PAP10c*	8	470	54.6	29, SRG-GT	2	23, 7-29	SP	
**BnaA09g08700D**	*BnaA9.PAP10*	8	468	54.1	29, CHG-GR	3	No	SP	
**BnaA07g15630D**	*BnaA7.PAP10*	7	325	37.8	Other	3	No	mTP	
**BnaA03g39100D**	*BnaA3.PAP10a*	7	466	53.8	25, CNG-GI	5	23, 7-29	SP	
**BnaA03g39080D**	*BnaA3.PAP10b*	8	426	49.4	Other	2	No	other	
**BnaA09g09490D**	*BnaA9.PAP11*	7	467	52.8	23, SHA-GV	3	No	SP	*AtPAP11* *AT2G18130* (H Block)
**BnaA07g01870D**	*BnaA7.PAP11*	7	459	52.1	24, SHA-GV	3	No	SP	
**BnaC09g09650D**	*BnaC9.PAP11*	7	467	52.8	23, SHA-GV	2	No	SP	

**BnaC04g38420D**	*BnaC4.PAP12a*	7	469	54	28, CDG-GI	3	20, 12-31	SP	
**BnaC04g38390D**	*BnaC4.PAP12b*	8	481	55.2	28, CDG-GI	3	23, 13-35	SP	*AtPAP12* *AT2G27190*
**BnaA04g15430D**	*BnaA4.PAP12a*	7	469	54.1	28, CDG-GI	3	20, 12-31	SP	(I Block)
**BnaA04g15420D**	*BnaA4.PAP12b*	8	481	55.5	28, CDG-GI	4	23, 13-35	SP	
**BnaC03g18120D**	*BnaC3.PAP13*	6	541	61.2	30, VDA-FP	5	23, 13-35	SP	*AtPAP13* *AT2G32770*
**BnaA03g15100D**	*BnaA3.PAP13*	7	534	60.2	27, VDA-FP	6	23, 7-29	SP	(J Block)
**BnaA05g01020D**	*BnaA5.PAP14*	5	393	44.5	24, VDA-YG	1	No	SP	*AtPAP14* *AT2G46880* (J Block)
**BnaUnng02540D**	*BnaUn.PAP15*	6	541	60.5	30, TFS-DA	8	23, 7-29	SP	
**BnaAnng39640D**	*BnaAn.PAP15*	5	383	43.3	Other	5	No	other	*AtPAP15* *AT3G07130*
**BnaC05g44840D**	*BnaC5.PAP15*	5	540	61.3	26, VSA-DY	4	No	SP	(F Block)
**BnaA05g30450D**	*BnaA5.PAP15*	6	521	59.1	26, SSA-DY	4	No	SP	
**BnaAnng16610D**	*BnaAn.PAP16*	4	384	42.9	23, AVG-WE	1	23, 7-29	SP	*AtPAP16* *AT3G10150*
**BnaC05g42920D**	*BnaC5.PAP16*	4	401	45	Other	1	23, 24-46	cTP	(F Block)
**BnaC05g35720D**	*BnaC5.PAP17*	3	337	38.3	Other	1	23, 7-29	SP	
**BnaC01g34650D**	*BnaC1.PAP17*	3	333	37.8	26, TNG-EL	1	No	SP	*AtPAP17* *AT3G17790*
**BnaA05g22460D**	*BnaA5.PAP17*	3	337	38.2	Other	1	23, 7-29	SP	(F Block)
**BnaA01g27810D**	*BnaA1.PAP17*	3	333	37.8	26, TNG-EL	1	No	SP	
**BnaCnng46140D**	*BnaCn.PAP18*	5	435	49.6	19, VAA-DD	0	No	SP	*AtPAP18* *AT3G20500*
**BnaA05g20440D**	*BnaA5.PAP18*	5	435	49.7	19, VAA-DD	0	No	SP	(F Block)
**BnaC08g23980D**	*BnaC8.PAP2*	6	426	48.4	21, VSS-YD	2	No	SP	*AtPAP20*

	0								*AT3G52780*
**BnaA09g33190D**	*BnaA9.PAP20*	6	426	48.3	21, VSS-YD	1	18, 5-22	SP	(N Block)
**BnaC06g14110D**	*BnaC6.PAP21*	6	436	50.5	22, SQA-YN	1	No	SP	*AtPAP21* *AT3G52810* (N Block)
**BnaA07g15780D**	*BnaA7.PAP21*	6	439	50.9	22, SQA-YN	1	No	SP	
**BnaA09g33200D**	*BnaA9.PAP22*	6	447	51	35, SQA-DV	2	No	SP	*AtPAP22* *AT3G52820* (N Block)
**BnaC08g08870D**	*BnaC8.PAP23*	8	579	64.5	31, AGG-ES	2	23, 7-29	SP	*AtPAP23* AT4G13700 (I Block)
**BnaA08g05220D**	*BnaA8.PAP23*	7	547	61.1	31, AGG-ES	4	23, 7-29	SP	
**BnaC01g16730D**	*BnaC1.PAP24*	11	618	69.5	21, CSG-DA	1	No	SP	*AtPAP24* *AT4G24890* (U Block)
**BnaA01g14240D**	*BnaA1.PAP24*	11	617	69.6	24, GHA-SN	1	No	SP	
**BnaC08g08080D**	*BnaC8.PAP26*	9	475	55	22, GEG-GI	1	No	SP	*AtPAP26* AT5G34850 (S Block)
**BnaA08g07270D**	*BnaA8.PAP26*	9	480	55.4	27, GEG-GI	1	No	SP	
**BnaC09g53400D**	*BnaC9.PAP28*	4	399	45	Other	2	20, 13-32	SP	*AtPAP28* *AT5G57140* (W Block)
**BnaC02g11790D**	*BnaC2.PAP28*	6	330	37.1	Other	2	20, 13-32	SP	
**BnaA10g11170D**	*BnaA10.PAP28*	4	403	45.3	Other	3	20, 13-32	SP	
**BnaCnng15730D**	*BnaCn.PAP29*	3	380	42.6	27, TSS-HR	2	18, 9-26	SP	AtPAP29 AT5G63140 (X Block)
**BnaC03g51100D**	*BnaC3.PAP29a*	4	357	39.3	30, ASA-QS	3	18, 13-30	SP	
**BnaC03g51090D**	*BnaC3.PAP29b*	3	423	47.1	Other	3	No	SP	
**BnaA09g06490D**	*BnaA9.PAP29*	3	385	42.9	32, TSA-HR	2	23, 7-29	SP	
**BnaA06g22560D**	*BnaA6.PAP29a*	3	381	42.3	31, ASA-QG	1	23, 7-29	SP	
**BnaA06g22550D**	*BnaA6.PAP2*	3	302	33.3	Other	0	No	other	

9b	

Table S3. The tandemly duplicated genes detected in the *PAP* family genes in *Brassica napus*.

Tandemly Duplicated Genes	Chromosome	Start	Stop	Strand	Group
BnaC7.PAP7a	chrC07	27.944124	27.959603	-	IIb
BnaC7.PAP7b	chrC07	27.92906	27.936546	-	IIb
BnaC4.PAP12a	chrC04	39.622943	39.625372	+	Ia-1
BnaC4.PAP12b	chrC04	39.590792	39.593446	+	Ia-1
BnaC3.PAP10a	chrC03	31.319691	31.322131	-	Ia-1
BnaC3.PAP10b	chrC03	31.22143	31.225213	+	Ia-1
BnaC3.PAP10c	chrC03	31.187781	31.190218	-	Ia-1
BnaC3.PAP29a	chrC03	35.640273	35.642092	-	IIIb
BnaC3.PAP29b	chrC03	35.6243	35.626272	-	IIIb
BnaA6.PAP29a	chrA06	15.78	15.78	+	IIIb
BnaA6.PAP29b	chrA06	15.77	15.77	+	IIIb
BnaA4.PAP12a	chrA04	12.812717	12.815098	+	Ia-1
BnaA4.PAP12b	chrA04	12.807213	12.809846	+	Ia-1
BnaA3.PAP10a	chrA03	19.48	19.48	-	Ia-1
BnaA3.PAP10b	chrA03	19.46	19.46	+	Ia-1

Table S4. Normalized FPKM of detected *BnaPAPs* in RNA-seq analysis.

Gene name	PsL	PdL	PsR	PdR
BnaA1.PAP24	1.69985	14.5417	2.59113	14.4927
BnaA1.PAP17	1.99363	132.906	0.267201	79.1719
BnaA2.PAP7	0.00617607	1.50689	0.314121	6.44428
BnaA3.PAP13	1.07961	0.59048	0.745992	0.736354
BnaA3.PAP10b	0.179232	2.58452	0.295683	1.31265

BnaA3.PAP10a	6.14502	5.13259	7.16658	7.41834
BnaA4.PAP12b	4.78475	8.76375	1.91075	4.30027
BnaA4.PAP12a	10.0927	78.4004	4.87312	68.9121
BnaA5.PAP14	0.0165214	48.8863	0.050429	0.132193
BnaA5.PAP18	7.03393	9.95148	22.5848	21.4412
BnaA5.PAP17	0.0502397	104.567	0.118125	40.3173
BnaA5.PAP15	1.09056	2.26578	1.72352	2.27764
BnaA6.PAP1	0.301245	0.319483	4.34041	5.22467
BnaA6.PAP2	0	0	0.00608875	0
BnaA6.PAP29b	0.00931426	0.0595981	1.92366	11.0809
BnaA6.PAP29a	7.31582	18.5494	3.62013	1.58951
BnaA6.PAP8	7.75488	85.6361	2.44403	7.51835
BnaA6.PAP7	0.113812	56.6391	3.41739	31.6762
BnaA7.PAP11	0.00629906	1.84239	0	0.23552
BnaA7.PAP10	2.26132	9.19634	1.79803	29.0597
BnaA8.PAP23	2.0061	2.27034	4.46953	5.08538
BnaA8.PAP26	22.6665	31.757	13.8524	16.3774
BnaA8.PAP6	0	0.347626	0	0.0442995
BnaA8.PAP2	4.42329	6.44749	10.0942	10.6415
BnaA9.PAP29	17.6212	33.3112	13.6884	11.4287
BnaA9.PAP10	3.37389	55.8001	15.0676	173.561
BnaA9.PAP11	0	0.0355669	0.0198537	0.203075
BnaA9.PAP20	0	0	0.0092886	0.132022
BnaA9.PAP22	0.272586	29.3202	1.41536	63.2531
BnaA9.PAP2	7.01418	11.1218	13.0884	13.5013
BnaA9.PAP1	3.84297	47.9405	2.41689	31.5086
BnaA10.PAP28	1.26603	1.1178	4.53252	3.51141
BnaC1.PAP24	2.10777	8.90056	4.07297	9.30693
BnaC1.PAP17	0.66177	162.518	0.944901	70.69
BnaC2.PAP7	0.0247043	0.8288	7.75418	13.7604
BnaC2.PAP9	0.829377	2.06239	3.24949	4.19528
BnaC3.PAP13	0.315703	0.288387	0.306983	0.225154
BnaC3.PAP10b	0.0039165	0.0240238	0.161897	0.245365
BnaC3.PAP10a	7.54006	9.2416	4.33752	5.12073

BnaC3.PAP29b	56.0062	66.2704	38.9081	25.2566
BnaC3.PAP29a	0.0537327	0.389174	1.52323	8.91086
BnaC3.PAP2	2.41228	4.01552	5.92573	6.08817
BnaC3.PAP6	0	0.0421608	0	0.281656
BnaC4.PAP12b	6.90502	17.8613	0.807976	2.06289
BnaC4.PAP12a	7.39778	53.1654	3.8766	60.2958
BnaC5.PAP1	0.0457935	0	0.0511575	0.137949
BnaC5.PAP2	0.0261548	0.0578608	0.0421194	0.0460956
BnaC5.PAP17	0.134316	176.956	0.136498	111.018
BnaC5.PAP16	0.285698	1.10653	0.0493895	0.478648
BnaC5.PAP15	1.31206	2.79879	3.09999	4.82061
BnaC7.PAP7b	1.57884	1.37351	0.933054	3.03108
BnaC7.PAP7a	1.60049	13.428	9.00566	28.0415
BnaC7.PAP8	9.32224	37.4375	1.61643	3.24584
BnaC8.PAP26	29.8328	41.1121	58.6711	49.7928
BnaC8.PAP23	5.53817	17.4553	16.5239	28.8331
BnaC8.PAP3	15.3025	1.80329	1.7742	1.39183
BnaC8.PAP20	0.132501	0.0948829	0.0507433	0.314119
BnaC8.PAP2	3.85658	5.99269	10.1333	9.35243
BnaC8.PAP1	2.46733	21.9941	3.51272	15.6405
BnaC9.PAP10	0.196497	2.90759	0.788766	14.6434
BnaC9.PAP11	0	0	0	0
BnaA7.PAP21	0	0	0	0
BnaC6.PAP21	0	0	0	0
BnaC2.PAP28	0	0	0	0
BnaC3.PAP10c	0	0	0	0

Table S5. Transcripts Per Million (TPM) data of detected *BnaPAPs* in RNA-seq analysis.

GeneID	CK-S	Nacl-S	CK-R	NaCl-R
BnaC8.PAP1	3.01	4.07	0.886667	0.843333
BnaC5.PAP1	0.0366666666667	0.09	0.05	0
BnaA9.PAP1	2.316666667	2.423333333	6.383333	17.02333
BnaA6.PAP1	0	0	0.27	0.26
BnaC8.PAP2	11.21333333	10.15666667	4.473333	9.94

BnaC5.PAP2	0.02	0	0.03	0
BnaC3.PAP2	6.143333333	5.52	2.8	6.636667
BnaA9.PAP2	16.44666667	14.11333333	7.706667	14.68333
BnaA8.PAP2	11.25666667	10.30666667	6.803333	10.88667
BnaA6.PAP2	0	0	0	0
BnaA8.PAP3	8.13	6.513333333	69.69	74.29
BnaC8.PAP3	6.85	4.216666667	20.30333	11.42333
BnaCn.PAP5	0	0	0	0
BnaC7.PAP6	0	0	0	0
BnaA3.PAP6	0	0	0	0
BnaC3.PAP6	0	0	0	0
BnaA8.PAP6	0	0	0	0
BnaC7.PAP7a	20.45	7.81	1.006667	4.093333
BnaC7.PAP7b	0.646666667	2.38	1.356667	1.713333
BnaC2.PAP7	11.74333333	8.456666667	0	0.576667
BnaA6.PAP7	4.676666667	4.87	0.233333	2.456667
BnaA2.PAP7	0.393333333	0.893333333	0	0.0233333333333
BnaC7.PAP8	3.096666667	5.506666667	21.7	46.89
BnaA6.PAP8	3.216666667	11.56333333	8.196667	74.65667
BnaA2.PAP9	2.51	2.43	0.636667	1.646667
BnaC2.PAP9	3.556666667	3.03	0.613333	1.533333
BnaCn.PAP10	0	0.0333333333333	2.73	1.113333
BnaC9.PAP10	10.31333333	7.33	4.39	3.39
BnaC3.PAP10a	5.063333333	7.533333333	10.56	16.92667
BnaC3.PAP10b	0.246666667	0.426666667	0	0.193333
BnaC3.PAP10c	0	0	0	0
BnaA9.PAP10	17.03	11.15666667	1.336667	2.343333
BnaA7.PAP10	9.233333333	5.956666667	0.276667	0.916667
BnaA3.PAP10a	5.413333333	13.06	10.50333	19.75
BnaA3.PAP10b	0.763333333	1.236666667	1.69	2.136667
BnaA9.PAP11	0	0	0	0
BnaA7.PAP11	0	0	0	0
BnaC9.PAP11	0	0	0	0.0133333333333

BnaC4.PAP12a	3.17	4.813333333	5.376667	5.916667
BnaC4.PAP12b	0.22	0.42	4.263333	14.66
BnaA4.PAP12a	6.523333333	9.36	12.66	22.70667
BnaA4.PAP12b	1.053333333	2.133333333	5.266667	12.72
BnaC3.PAP13	0.07	0.156666667	0.353333	0.29
BnaA3.PAP13	0.366666667	0.26	0.75	0.716667
BnaA5.PAP14	0	0	0.0166666666667	0
BnaUn.PAP15	1.766666667	9.2	0.31	0.616667
BnaAn.PAP15	0.233333333	0.94	0.153333	0.293333
BnaC5.PAP15	1.006666667	1.236666667	0.816667	1.153333
BnaA5.PAP15	1.06	1.593333333	0.953333	1.84
BnaAn.PAP16	0.176666667	0.233333333	19.97667	26.51667
BnaC5.PAP16	0.0366666666667	0.0433333333333	0.78	0.74
BnaC5.PAP17	0.0133333333333	0.0133333333333	0.0733333333333	0.146667
BnaC1.PAP17	0.556666667	6.126666667	1.28	1.786667
BnaA5.PAP17	0.04	0.16	0	0
BnaA1.PAP17	0.413333333	5.513333333	1.85	1.38
BnaCn.PAP18	42.22666667	31.52666667	15.01333	39.83
BnaA5.PAP18	34.48	30.67	11.15	30.00333
BnaC8.PAP20	0.0633333333333	0.133333333	0.33	0.0266666666667
BnaA9.PAP20	0	0.51	0	0.22
BnaC6.PAP21	0	0	0.0166666666667	0
BnaA7.PAP21	0	0	0	0
BnaA9.PAP22	1.63	2.106666667	0.203333	13.34667
BnaC8.PAP23	13.61	11.17666667	4.766667	9.22
BnaA8.PAP23	5.99	5.336666667	2.38	4.843333
BnaC1.PAP24	1.18	1.716666667	0.36	1.156667
BnaA1.PAP24	4.133333333	4.933333333	2.106667	5.283333
BnaC8.PAP26	43.02	46.93	35.26667	71
BnaA8.PAP26	2.856666667	3.803333333	7.073333	17.34333
BnaC9.PAP28	6.643333333	5.556666667	1.733333	3.213333

BnaC2.PAP28	0	0	0	0
BnaA10.PAP28	0.976666667	0.5	0.123333	0.416667
BnaCn.PAP29	4.56	5.33	19.42	16.96
BnaC3.PAP29a	3.896666667	0.546666667	0.02	0
BnaC3.PAP29b	14.27333333	12.77	31.8	20.96
BnaA9.PAP29	3.47	8.31	6.383333	17.02333
BnaA6.PAP29a	3.01	2.583333333	4.346667	5.663333
BnaA6.PAP29b	5.6	1.696666667	0	0.0666666666667

Table S6. Number and position of *cis*-regulatory elements related to Pi stress present in the promoters of *PAP* genes in *B.napus*.

Genes	Group	NO. of P1BS[a]	Position of P1BS[b]	NO. of W-Box[c]	Position of W-Box
BnaC9.PAP10	Ia-1	3	-145、-949、-978	2	-471 -1844
BnaC3.PAP10a	Ia-1	1	-377	2	-532 -831
BnaC3.PAP10b	Ia-1	0		2	-1482 -1450
BnaC3.PAP10c	Ia-1	3	-1297、-1902、-1610		
BnaA9.PAP10	Ia-1	3	-146、-1408、-1437		
BnaA7.PAP10	Ia-1	0		2	-1508、-1520
BnaA9.PAP11	Ia-1	1	-89	1	-937
BnaC9.PAP11	Ia-1	1	-89	1	-1056

BnaA7.PAP11	Ia-1	2	-89、-189	1	-1705
BnaC9.PAP11	Ia-1	0		1	-1057
BnaC4.PAP12a	Ia-1	1	-132	0	
BnaC4.PAP12b	Ia-1	1	-128	0	
BnaA4.PAP12a	Ia-1	1	-132	0	
BnaA4.PAP12b	Ia-1	0		0	
BnaC7.PAP6	Ia-1	2	-91、-117	2	-1549、-1624
BnaA3.PAP6	Ia-1	3	-88、-105、-1517	2	-1815、-1889
BnaA8.PAP6	Ia-1	2	-95、-248	2	-1213、-1272
BnaA8.PAP26	Ia-2	0		1	-996
BnaC8.PAP26	Ia-2	0		1	-1937
BnaA8.PAP23	Ib-1	0		2	-298 -1399
BnaC8.PAP23	Ib-1	1	-161		
BnaC5.PAP15	Ib-1	1	-885	1	-475
BnaUn.PAP15	Ib-1	0		0	
BnaA5.PAP15	Ib-1	1	-359	1	-503
BnaCn.PAP18	Ib-2	2	-735、-1580	2	-296 -1618
BnaA5.PAP18	Ib-2	2	-898、-1734	2	-475 -1772
BnaC8.PAP20	Ib-2	0		3	-636 -896 -1864
BnaC6.PAP21	Ib-2	1	-1486		
BnaC8.PAP1	Ic-1	1	-195	0	
BnaA9.PAP1	Ic-1	2	-207、-1337	2	-1156、-1242
BnaA6.PAP1	Ic-1	1	-398	0	
BnaA1.PAP24	Ic-1	1	-1135		
BnaC1.PAP24	Ic-1	0		1	-206
BnaA2.PAP9	Ic-2	1	-1038	2	-30 -787
BnaC2.PAP9	Ic-2	0		2	-30 -792
BnaC3.PAP2	Ic-2	0		1	-1705
BnaA9.PAP2	Ic-2	0		1	-466
BnaC5.PAP2	Ic-2	0		1	-1808
BnaA6.PAP8	IIa	0		1	-74
BnaC7.PAP8	IIa	0		0	
BnaC5.PAP17	IIa	3	-284、-326、-1084	1	-1820
BnaC1.PAP17	IIa	3	-147、-447、-597	2	-1435 -1939
BnaA5.PAP17	IIa	2	-579、-634	0	
BnaA1.PAP17	IIa	4	-155、-465、-615、-1974	1	-1766
BnaA8.PAP3	IIa	0		2	-882、-946
BnaC8.PAP3	IIa	0		1	-303
BnaC7.PAP7a	IIb	1	-1408	2	-849、-1963

BnaC2.PAP7	IIb	2	-390、-467	0	
BnaA2.PAP7	IIb	0		1	-1883
BnaA6.PAP7	IIb	2	-1268、-1342	0	
BnaAn.PAP16	IIIa	2	-323、-349	2	-881 -1422
BnaC5.PAP16	IIIa	3	-249、-276、-646	2	-437 -1811
BnaA5.PAP14	IIIb	4	-128、-170、-238、-352	2	-935 -1256
BnaC2.PAP28	IIIb	0		2	-963 -1756
BnaA10.PAP28	IIIb	0		3	-671 -1576 -1836
BnaA9.PAP29	IIIb	0		3	-1059 -1579 -1674
BnaC3.PAP29b	IIIb	0		4	-1117 -1126 -1133 -1141
BnaCn.PAP29	IIIb	0		3	-1061 -1578 -1673
BnaA6.PAP29a	IIIb	0		3	-179 -187 -680
BnaA6.PAP29b	IIIb	3	-86、-464、-1991	1	-162

Table S7 Primers used in the present study.

Primer name	Forward sequence (5' -3')	Reverse sequence (5' -3')
Primers used in vector construction		
pBnaC9.PAP10:GUS	aagcttCTGGTATGATCAGTGGTCTTC	cccgggCATTTTCTCAGCTTCTCTTATC
pBnaC9.PAP10ΔW:GUS-P1	aagcttGGAAATTATGGGACTACGG	GGAATATAATAGTATTGAAAACAAAATGAAGAAGG
pBnaC9.PAP10ΔW:GUS-P2	CCTTCTTCATTTTGTTTTCAATACTATTATATTCCTTC	cccgggCATTTTCTCAGCTTCTCTTATC
pBnaC9.PAP10ΔWΔP:GUS-P1	aagcttCTTGCTTTGTGATCTACG	CTTAAGAACCCGTACGAAGTTGCCAACTC
pBnaC9.PAP10ΔWΔP:GUS-P2	CAACTTCGTACGGGTTCTTAAGTTAACTC	cccgggCATTTTCTCAGCTTCTCTTATC
pBnaA7.PAP10:GUS	gtcgacCATGTATCTATAAAATAATTCTG	cccgggTCATGGCCTTTGAAGTC
pBnaA7.PAP10ΔW:GUS	gtcgacGGTTCGGATCAGACTGGC	cccgggTCATGGCCTTTGAAGTC
Primers used in RT-qPCR analysis		
BnaActin2	ACAGTGTCTGGATCGGTGGTTC	TGCCTCATCATACTCAGCCTTG
qBnaA5.PAP17	GATTATAGAGGTGACTCAGGC	GAGGAGAGACTTGACATAAGAG
qBnaC5.PAP17	TCGCCGCGGTGACTTCAAC	GACTTGGAGCAGTGTAGATATC
qBnaA9.PAP1	GTCTTGGTGACTCTTTATGTAG	GTCTGATGGGTGTGGAGAAG
qBnaC8.PAP1	GGGATGTTTAGGTTCTGTGTG	GCCACACAGAACCTAAACATC
qBnaA7.PAP10	CTTCAGGCAGCACAGAACC	AACATGTAATGGTAATCGTAAC

qBnaC9.PAP10	AAGATAAATCTGCTCCAGTC	GTCCTGTTCTTTATCGATAAC
qBnaA4.PAP12a	CTACAACCTTGTCAATGGG	CAGTTGAAGTAGGCATGAGTT
qBnaC4.PAP12a	TATCAAGAGGGCTTCAGCTTAC	AAGGGGCAATGTACTAAGACC
qBnaA1.PAP24	GTTCGAACCTATGTTGCGGTC	CTGCGATCCAATCGTTTGAAC
qBnaC1.PAP24	GAGTTCGAGATGTTCGGGTG	CAACAGGTAGATGAGTCGAACG
qBnaA8.PAP26	GATGGAAAGGCAGTTATCGTG	ATTATACTCAAGGCCAGAGAGA
qBnaC8.PAP26	GATGGAAAGGCTGTTATCATC	ATTATACTCAAGGCCAGAGACT
